# Asymmetric Conjugate Hydrocyanation of *α*,*β*‐Unsaturated Aldehydes Catalyzed by Engineered 2‐Deoxy‐D‐ribose‐5‐phosphate Aldolase

**DOI:** 10.1002/chem.202503435

**Published:** 2025-12-12

**Authors:** Hangyu Zhou, Peter Fodran, Haigen Fu, Gerrit J. Poelarends

**Affiliations:** ^1^ Department of Chemical and Pharmaceutical Biology, Groningen Research Institute of Pharmacy University of Groningen Groningen the Netherlands; ^2^ NHC Key Laboratory of Biotechnology for Microbial Drugs, Institute of Medicinal Biotechnology Chinese Academy of Medical Sciences and Peking Union Medical College Beijing China

**Keywords:** aldolase, biocatalysis, catalytic promiscuity, hydrocyanation, nitriles

## Abstract

The enantioselective conjugate hydrocyanation of *α*,*β*‐unsaturated aldehydes remains a long‐standing challenge in synthetic chemistry. Here, we report the redesign of 2‐deoxy‐D‐ribose‐5‐phosphate aldolase (DERA) into an efficient biocatalyst capable of promoting the asymmetric conjugate addition of hydrogen cyanide (generated in situ from trimethylsilyl cyanide) to aromatic enals via an iminium activation pathway. The evolved variant DERA‐CN enables the efficient formation of various C4‐nitriles with high conversions (up to 99%) and good enantioselectivity (up to 98% e.e.). Control experiments revealed a stepwise process involving enzyme‐catalyzed conjugate hydrocyanation followed by spontaneous 1,2‐addition of cyanide. Substrates with various electron‐donating and electron‐withdrawing groups are tolerated, providing access to various enantioenriched nitriles. This work expands the scope of DERA‐promoted iminium catalysis and provides a rare enzymatic platform for asymmetric conjugate hydrocyanation under mild aqueous conditions.

## Graphical abstract

The power of catalytic promiscuity: The enantioselective conjugate hydrocyanation of enals remains a long‐standing challenge for biocatalysis. Here, we report the redesign of 2‐deoxy‐D‐ribose‐5‐phosphate aldolase for the asymmetric conjugate hydrocyanation of aromatic enals, expanding the reaction scope of iminium‐based enzyme catalysis to include an additional new‐to‐nature reaction.

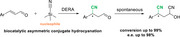

## 1 Introduction

2‐Deoxy‐d‐ribose‐5‐phosphate aldolase (DERA), a class I aldolase, naturally catalyzes the reversible aldol addition between acetaldehyde (aldol donor) and d‐glyceraldehyde‐3‐phosphate (aldol acceptor) [1]. The lysine residue (Lys 167) located in the active site plays a pivotal catalytic role: its *ε*‐amino group activates the aldol donor via enamine formation, thereby initiating the aldol reaction [2, 3]. Building on its mechanistic understanding, we have demonstrated that DERA can be redesigned into a proficient “Michaelase” that catalyzes the enantioselective Michael addition of nitromethane to *α*,*β*‐unsaturated aldehydes [4], unlocking its potential for iminium catalysis [5, 6, 7], which is distinct from its native enamine catalysis activity. Following this pioneering work, the interest in discovering new reactivity of DERA based on the iminium activation mode has increased spectacularly. For example, Tseliou et al. have revealed that DERA was able to function as a nonnatural photodecarboxylase, promoting stereospecific radical coupling of appropriate radical donor substrates, providing a rare example of photobiocatalysis via direct visible‐light excitation of enzyme‐bound catalytic intermediates [8]. Similar enantioselective radical coupling via DERA‐catalyzed photodecarboxylation was discovered by Saravanan and coworkers [9]. More recently, we have evolved DERA into a peroxygenase capable of catalyzing the enantioselective epoxidation of *α*,*β*‐unsaturated aldehydes using hydrogen peroxide as nucleophile [10], further underscoring the functional plasticity of DERA for iminium catalysis.

**FIGURE 1:**
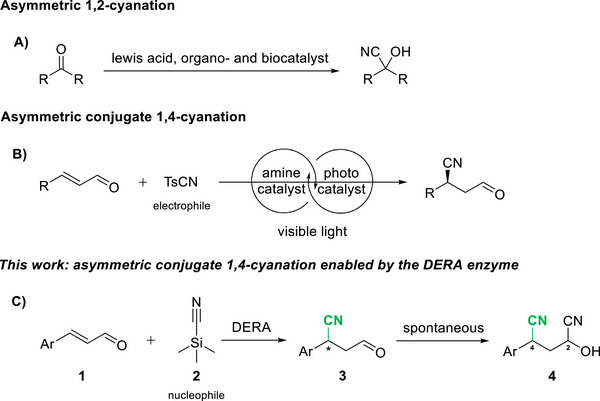
Asymmetric cyanation of aldehydes. (A) Asymmetric 1,2‐cyanation of enals catalyzed by Lewis acid, organo‐, and biocatalysts. (B) Asymmetric conjugate 1,4‐cyanation of enals by combining photoredox‐ and organocatalysis. (C) DERA catalyzed asymmetric conjugate 1,4‐cyanation (nucleophilic HCN derived in situ from trimethylsilyl cyanide **2**) of *α*,*β*‐unsaturated aldehydes **1**.

Motivated by these findings, we aimed to identify other nucleophiles that can be accepted by DERA in conjugate addition reactions using *α*,*β*‐unsaturated aldehydes as electrophiles. Cyanide was of particular interest given its small, linear geometry and high nucleophilicity. Asymmetric 1,2‐addition of cyanide to aldehydes has been intensively explored, driven by the broad utility of enantiomerically enriched nitriles in natural products, and medicinal and agrochemical synthesis [11, 12, 13, 14]. Their synthetic versatility lies in the fact that the nitrile group can be readily converted into a variety of functional groups—including carboxylic acids, amines, amides, and others—via well‐established chemical and enzymatic methods, thereby offering strategic flexibility in complex molecule construction [15]. Lewis acid catalysts [16], organocatalysts [17], and biocatalysts, particularly hydroxynitrile lyases (HNLs) [18, 19], enable efficient asymmetric 1,2‐cyanation of aldehydes to access enantioenriched cyanohydrins (Figure 1A).

In contrast, the chemoselective conjugate 1,4‐cyanation of enals is still underdeveloped; the main difficulty lies in accomplishing 1,4‐chemoselectivity over the preferred 1,2‐addition of cyanide. A notable exception involves the synergistic use of a chiral organocatalyst and a visible‐light‐activated photoredox catalyst, which inverts the innate reactivity of enals through an umpolung mechanism via single‐electron reduction. The resultant chiral radical is then captured by an electrophilic cyanide source with 1,4‐chemoselectivity and good stereocontrol (Figure 1B) [20]. Despite this ground‐breaking advance, an effective enzymatic method for chemo‐ and enantioselective 1,4‐cyanation of enals has not been established to date. Herein, we report the redesign of DERA to achieve asymmetric conjugate hydrocyanation of various *α*,*β*‐unsaturated aldehydes **1**, using trimethylsilyl cyanide **2**, affording the corresponding nitriles with excellent enantiopurity (Figure 1C). Upon addition to the aqueous reaction solution, trimethylsilyl cyanide **2** undergoes hydrolysis to release hydrogen cyanide in situ, which then serves as the nucleophile. The use of **2** as a hydrogen cyanide donor offers practical advantages due to its reduced toxicity and volatility compared to free hydrogen cyanide [21, 22, 23].

## 2 Results and Discussion

**FIGURE 2:**
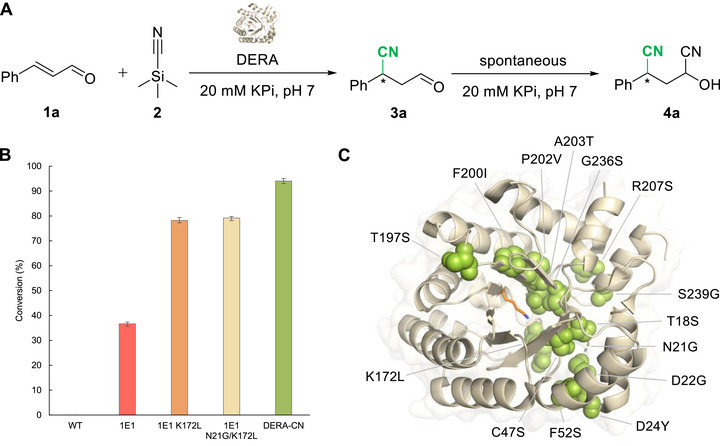
Hydrocyanation activity of DERA variants. A) Reaction of the DERA catalyzed hydrocyanation of cinnamaldehyde **1a** using **2** to afford **3a**; notably, the enzymatic product **3a** undergoes spontaneous 1,2‐cyanation to yield **4a**. B) Comparison of the catalytic activity of wild‐type DERA and engineered DERA variants for the hydrocyanation reaction. Analytical scale reactions were performed with 1 mM **1a**, 20 mM **2**, and 50 µM DERA variants in 20 mM KPi, pH 7, 7.5% v/v EtOH at room temperature, reaction volume = 400 µL, reaction time = 18 h. Conversion was determined by GC‐FID based on the calibration curve using mesitylene as the internal standard. Error bars represent the deviation of measurements made in duplicate. C) Overall structure of DERA‐CN shown in a cartoon representation. The key catalytic residue, lysine 167, is shown as an orange stick. Mutations present in DERA‐CN are indicated as green spheres.

We started our investigation by evaluating whether wild‐type DERA, DERA‐MA (the best‐performing variant for Michael additions) [4], or DERA‐EP (the best‐performing variant for epoxidations) [10] can promiscuously catalyze the hydrocyanation of cinnamaldehyde (**1a**) with trimethylsilyl cyanide **2** to give the corresponding product **3a**. When 240 µM enzyme was incubated with 1 mM **1a** and 20 mM **2** at room temperature, both DERA‐MA and DERA‐EP displayed hydrocyanation activity. Complete conversion of substrate **1a** into product was observed with DERA‐EP, whereas only trace amounts of product were detected with DERA‐MA, and no product formation was detected with the wild‐type enzyme (Figure S1). Upscaling the experiment with DERA‐EP (see Supporting Information for details) afforded enough material for a complete structural analysis by HRMS, ^1^H NMR, ^13^C NMR, COSY, HSQC, and HMBC, which identified the final product as 2‐hydroxy‐4‐phenylpentanedinitrile **4a**, a compound bearing nitrile groups at both the C2 and C4 positions (Figure S5). A control reaction under otherwise similar conditions but without the enzyme did not yield product **4a**, confirming the essential catalytic role of the enzyme. An additional control experiment without the presence of the enzyme showed that aldehyde **3a** was also converted into product **4a**, confirming that the 1,2‐cyanation of **3a** occurs spontaneously (Table S6). Chiral normal‐phase HPLC analysis revealed that DERA‐EP achieves high stereocontrol at C4, giving product **4a** with an enantiomeric excess (e.e.) of 80%. However, the spontaneous cyanation of **3a** resulted in a poor diastereomeric ratio (d.r. = 55:45). Hence, the active site of DERA‐EP can give rise to synthetically useful catalytic promiscuity, promoting chemoselective 1,4‐addition of cyanide to cinnamaldehyde to form enantioenriched product **3a**, which then undergoes a spontaneous 1,2‐addition of cyanide to furnish the final dinitrile product **4a** (Figure 2A).

Given the low hydrocyanation activity of DERA‐EP, we applied an engineering approach to generate more active variants (Table S1). Screening a previously constructed library of DERA variants led us to identify five variants (DERA 9610C8, 1E1, 2A7, 2B9, and 9F11) with similar hydrocyanation activity as DERA‐EP. Among these, DERA 9610C8 harbored the fewest mutations (10 mutations), with K172L as the only unique substitution relative to the others (11–14 mutations; Table S2). Hypothesizing that K172L has a significant influence on the hydrocyanation activity, we introduce this point mutation into the other 5 variants. To our delight, variant 1E1 K172L displayed outstanding hydrocyanation activity compared to the other K172L mutants. Subsequent site‐saturation mutagenesis at position 172 in the 1E1 variant revealed leucine as the optimal residue for catalytic enhancement. Furthermore, 4 residues (17, 18, 20, 21) located in the highly disordered *β*1‐*α*2 loop and 4 residues (169, 170, 171, 173) located in the *β*7‐*α*8 loop near the key catalytic residue Lys167 were targeted for site‐saturation mutagenesis. Activity screening of these 8 mutant libraries identified 1E1 N21G/K172L as the most active variant. Finally, a random mutagenesis strategy was applied on the basis of variant 1E1 N21G/K172L, which led to the discovery of the final variant DERA‐CN (1E1 N21G/K172L/F200I). Under standard conditions (1 mM **1a**, 20 mM **2**, room temperature), DERA‐CN consumed over 94% of substrate **1a** within 18 h (Figure 2B). Compared to wild‐type DERA, which showed no hydrocyanation activity, this engineered variant represents a significant breakthrough, transforming a previously inaccessible reaction into a viable catalytic process.

With this improved variant in hand, we turned to the question of whether the second stereogenic center at C2 could be introduced by a complementary enzyme such as hydroxynitrile lyase (HNL), which catalyzes the reversible addition of cyanide to the carbonyl group of aldehydes and ketones. By combining DERA‐CN and HNL, the diastereomeric outcome of product **4a** could potentially be enhanced. The hydroxynitrile lyase from *Arabidopsis thaliana* (*At*HNL) was tested in the envisioned cascade. However, co‐incubation of 20 µM DERA‐CN with 10 µM *At*HNL did not lead to any significant improvement in the diastereomeric ratio (Table S5, entry 1; Table S4, entry 1), suggesting that the nonenzymatic cyanation step proceeds significantly faster than the *At*HNL‐mediated 1,2‐addition of cyanide.

Since buffer composition and pH might influence the activity and selectivity of DERA‐CN and *At*HNL [24, 25], as well as the nonenzymatic cyanation reaction [26, 27], a series of buffers was evaluated (Table S4). Notably, the product diastereomeric ratio showed little variation with different buffers. Consequently, the low diastereoselectivity likely arises from the poor affinity of *At*HNL for aldehyde intermediate **3a** and the fast nonenzymatic 1,2‐addition of hydrogen cyanide. However, when 50 mM citrate buffer (pH 5.5) was applied the enantiomeric ratio resulting from the enzymatic construction of the C4 stereogenic center increased markedly from 85:15 to 99:1 (Table S4, entries 1 and 5).

**FIGURE 3:**
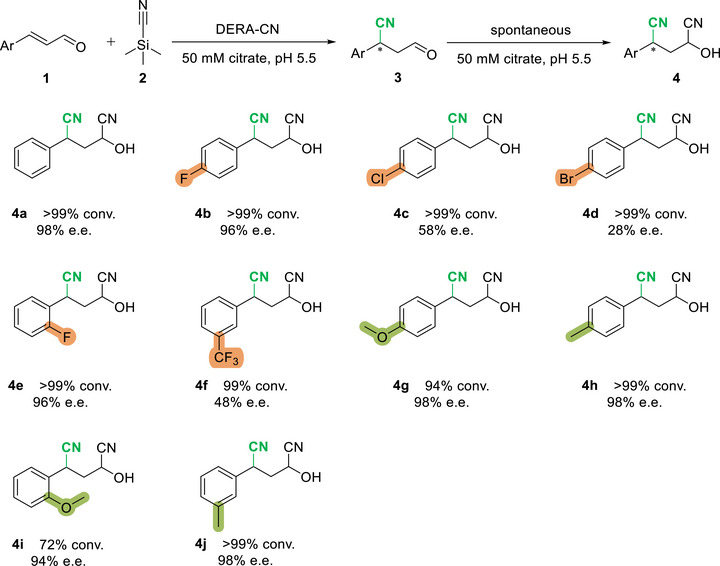
Substrate scope of DERA‐CN. Analytical scale reactions were performed with 1 mM **1a‐j**, 20 mM **2**, and 50 µM DERA‐CN in 50 mM citrate, pH 5.5, 7.5% v/v EtOH (ACN for **1d** and **1** **g**) at room temperature, reaction volume = 400 µL, reaction time = 24 h. Enzymatic product **3** undergoes spontaneous 1,2‐addition of hydrogen cyanide to form compound **4**. The diastereomeric purity of the products is provided in the Supporting Information. Conversions were determined by GC‐FID using mesitylene as an internal standard. The enantiomeric excess (e.e.) was determined by chiral normal‐phase HPLC.

Next, we investigated the substrate scope of DERA‐CN with various cinnamaldehyde derivatives bearing both electron‐withdrawing (F, Cl, Br, CF_3_) and electron‐donating (Me, OMe) substituents on the aryl ring in 50 mM citrate buffer (pH 5.5). As shown in Figure 3, all substrates (**1b‐1f**, **1** **h**, **1j**) except the OMe‐substituted aldehydes (**1** **g** and **1i**) were well accepted by DERA‐CN, with conversions exceeding 99%. Notably, the electron‐donating OMe group in the *ortho* position (**1i**) resulted in lower conversion (72%), while the same group in the *para* position (**1** **g**) resulted in high conversion (94%), both affording products with excellent enantiopurity. Aldehydes with an electron‐donating Me group at the *meta* or *para* position (**1** **h** and **1j**) gave excellent conversion (more than 99%) and product enantiopurity (e.e. more than 98%, Figure S25, S27). Among all the substitutions, the substrate bearing *p*‐Cl, *p*‐Br, or *m*‐CF_3_ group (**1c**, **1d,** and **1f**) delivered products with compromised enantiopurity (e.e., from 28% to 58%, Figure S20, S21, S23). Overall, high conversions (up to 99%) were achieved for a broad range of aromatic *α*,*β*‐unsaturated aldehydes (**1b‐1j**), furnishing the corresponding hydroxydinitrile products (**4b–4j**) in good to excellent enantiopurity (e.e. up to 98%, Figure S19–S27). These results underscore the utility of enzyme promiscuity for developing new biocatalysts for challenging new‐to‐nature reactions.

## 3 Conclusions

In conclusion, the recently discovered capability of DERA to adopt iminium catalysis prompted us to examine its ability to promote the conjugate hydrocyanation of *α*,*β*‐unsaturated aldehydes. While wild‐type DERA cannot catalyze this reaction, we have demonstrated the feasibility of enantioselective conjugate hydrocyanation of cinnamaldehydes using evolved DERA variants, expanding the reaction scope of iminium‐based enzyme catalysis to include an additional new‐to‐nature reaction. Through iterative rounds of rational design, site‐saturation mutagenesis, and random mutagenesis, we developed the DERA‐CN variant (1E1 N21G/K172L/F200I), which demonstrated good catalytic activity, broad substrate scope, and excellent enantioselectivity across a series of cinnamaldehyde derivatives.

Control experiments suggest a two‐step pathway involving asymmetric enzyme‐catalyzed 1,4‐addition of hydrogen cyanide (generated in situ from trimethylsilyl cyanide) followed by spontaneous 1,2‐addition to form valuable enantioenriched hydroxydinitrile products. Despite the stereochemical limitations posed by the nonenzymatic chemical reactivity in the second cyanation step, the simple reaction setup and the mild reaction conditions render this robust biocatalytic system an appealing process to produce important chiral synthons for the preparation of bioactive natural products and pharmaceuticals [23]. In future work, we aim to convert the enzymatic products **3** or chemoenzymatic products **4** into optically pure diastereomeric products using different enzymatic cascade approaches.

Importantly, this work establishes a rare example of biocatalytic conjugate hydrocyanation and lays the groundwork for the development of artificial enzyme cascades involving nucleophilic cyanide species. The respectable activity and enantioselectivity of DERA‐CN highlight the broader potential of enzyme engineering in unlocking new‐to‐nature reactivity, providing powerful tools for sustainable and chemo‐ and enantioselective chemical synthesis. Further investigations on exposing new carbonyl transformation activities in wild‐type DERA and engineered DERA variants are ongoing in our laboratory.

## 4 Experimental Section

### 4.1 Enzyme Activity Assay

The DERA cyanation activity assays were performed at room temperature. Fresh stock solutions of **1a** (40 mM in EtOH) and **2** (400 mM) were prepared. The reaction mixture (400 µL) consisted of **1a** (1 mM), **2** (20 mM), and DERA variant (50 µM) in 20 mM KPi (pH 6.5), or 50 mM citrate (pH 5.5), with 7.5% (v/v) EtOH (or ACN). For each enzymatic reaction, a negative control reaction without enzyme (enzyme replaced by buffer) was set up in parallel. After 18 h (or 24 h), the reaction mixture was used for extraction with EtOAc. Calibration curves of compound **1a** were obtained using 1 mM mesitylene as the internal standard. The GC‐FID data for the analytical scale reactions were used to determine the conversion. The enantiomeric ratio and diastereomeric ratio were determined by chiral normal phase HPLC analysis.

## Conflicts of Interest

The authors declare no conflict of interest.

## Supporting information




**Supporting file 1**: Detailed experimental methods, synthetic procedures, and full characterization data are included in the Supporting Information. The authors have cited additional references within the Supporting Information [28, 29, 30, 31, 32, 33, 34, 35].

## Data Availability

The data that support the findings of this study are available in the supplementary material of this article.
