# Supplementary material for: Asymmetric Conjugate Hydrocyanation of α,β‐Unsaturated Aldehydes Catalyzed by Engineered 2‐Deoxy‐D‐ribose‐5‐phosphate Aldolase
Source: Chemistry. 2025 Dec 12;32(2):e03435. doi: 10.1002/chem.202503435 (PMC12790325; doi:10.1002/chem.202503435)
Supplement: Supplementary file 1 — Supporting file 1: Detailed experimental methods, synthetic procedures, and full characterization data are included in the Supporting Information. The authors have cited additional references within the Supporting Information [28, 29, 30, 31, 32, 33, 34, 35]. [file CHEM-32-e03435-s001.pdf]

Supporting information

1. Materials and methods
2. Identification of a suitable starting point for evolution
3. Engineering overview
4. Site-directed mutagenesis
5. Site-saturation mutagenesis
6. Random mutagenesis by error-prone PCR
7. Screening
8. Enzyme expression and purification
9. Enzyme activity assay
10. Upscale synthesis of **4a** catalyzed by DERA-EP
11. Buffer optimization
12. Substrate scope analysis of DERA-CN
13. Synthesis of the racemic reference compounds **4**
14. DNA and protein sequences
15. NMR spectra
16. GC-FID analysis
17. Chiral normal phase HPLC analysis
18. Supplementary references

CAUTION: Trimethylsilyl cyanide is highly poisonous. Procedures involving trimethylsilyl cyanide were performed in a well-ventilated lab hood to ensure safety. Neutralization of HCN-containing wastes was performed with 5 M sodium hydroxide or potassium hydroxide solution.

1. Materials and methods

All oligonucleotide primers used in this study (summarized in **Table S3**) were purchased from Eurofins Genomics. Spin miniprep kit for plasmid isolation and PCR purification kit were purchased from QIAGEN. Q5 High-Fidelity 2× Master Mix was purchased from New England BioLabs. GoTaq® MDxHot start polymerase was purchased from Promega Corporation. *Dpn*I, *Nde*I and *Xho*I restriction endonuclease, T4 DNA ligase, protein ladders and precast electrophoresis gels for protein analysis were purchased from Thermo Fisher Scientific. Bugbuster for cell lysis was purchased from EMD Biosciences. AcroPrep™ Advance 96-well filter plate for protein purification was purchased from Pall Life Science. HisTrap™ HP chromatography columns for protein purification, PD-10 Sephadex™ G-25M columns for buffer exchange and PD MultiTrap™ G-25 for buffer exchange were purchased from Cytiva. 96-well microplate (F-bottom, 300 uL) and 96-well microplate (UV-star F-bottom, 300 uL) were purchased from Greiner Bio-one. InstantBlue® Coomassie Protein Stain was purchased from abcam. Other aldehyde substrates and chemicals were purchased from Sigma-Aldrich Chemical Co. (St. Louis, MO, USA), BLD Pharmatech Ltd. (Shanghai, China), TCI Europe N.V. (Zwijndrecht, Belgium) or Thermo Fisher Scientific (Geel, Belgium).

ESI-MS analysis of purified enzyme variants was performed by the Mass Spectrometry core facility of the University of Groningen. Spectrophotometric measurements were performed on a V-650 or V-660 spectrophotometer from Jasco (IJsselstein, The Netherlands), and measurements in a 96-well format were performed on a SPECTROstar Omega plate reader (BMG LABTECH, Isogen Life Science, de Meern, The Netherlands). Chiral reverse phase high performance liquid chromatography (HPLC) was performed with a Shimadzu LC-20AD HPLC with a Shimadzu SPD-M20A diode array detector or SPD-20A uv/vis detector. Chiral normal phase HPLC was performed with a Shimadzu LC-10AT HPLC with a Shimadzu SPD-M10A diode array detector. GC-MS was conducted with a Shimadzu GC-MS-QP2010 SE. NMR spectra were recorded on JEOL (NM-70010S4L1) NMR spectrometer. The chemical shifts (δ) are reported in parts per million (ppm). ^{1}H spectra are referenced to the shift of the residual protio solvent (7.26 for $CDCl_3$) and ^{13}C NMR spectra are referenced to shift of the deuterated solvent (77.16 for $CDCl_3$). Multiplicity are reported as singlet (s), doublet (d), triplet (t), quartert (q), doublet of doublets (dd), doublet of triplets (dt), triplet of (td), and multiplet (m) and coupling constants (*J*) are in Hz.

2. Identification of a suitable starting point for evolution

A collection of DERA variants was constructed previously[1,2] and used directly for the first round of activity screening.

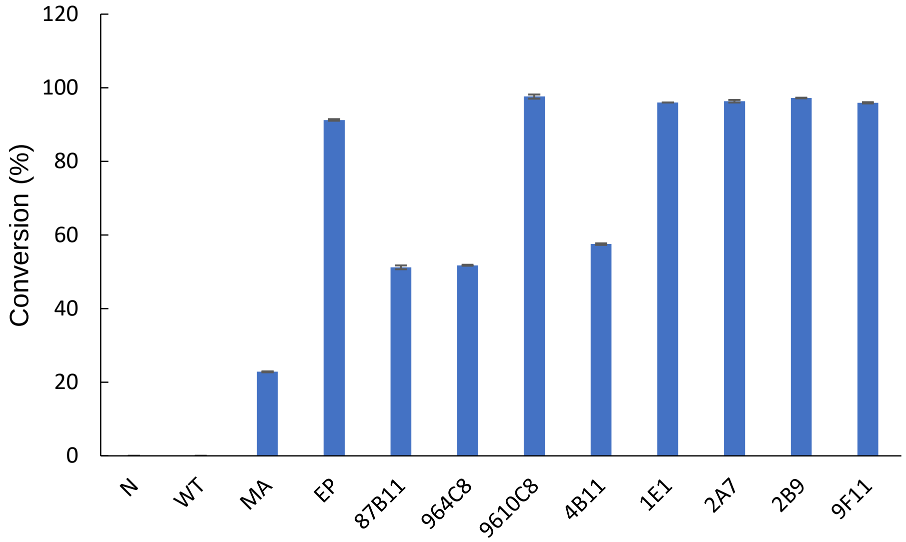

Figure S1. Identification of a suitable starting point for evolution. Conversion of cinnamaldehyde (**1**) to the corresponding cyanation product using trimethylsilyl cyanide (**2**) and different DERA variants. Reactions were performed with 1 mM **1**, 20 mM **2** and 240 µM DERA variant in 20 mM KPi, pH 7 with 7.5% v/v EtOH at room temperature for 19 h, reaction volume = 200 µL. Error bars represent the deviation of measurements made in duplicate. N: negative control, reaction without enzyme. WT: wild-type DERA.

3. Engineering overview

Table S1. Overview of the directed evolution of DERA towards enhanced cyanation activity.

Round	Input	Evolution strategy	Library size [a]	Output
1	wild-type DERA	screen DERA variants library[1,2]	11	EP, 9610C8, **1E1**, 2A7, 2B9, 9F11
2	EP, **1E1**, 2A7, 2B9, 9F11[b]	SDM (K172L)	5	**1E1 K172L**
3	**1E1**	SSM (position 172, NNK)	89 (coverage 93%)	**1E1 K172L**
4	1E1 K172L	SSM (8 positions, NNK) [c]	712 (coverage 93%)	**1E1 N21G/K172L**
5	1E1 N21G/K172L	error prone	1068	GL 1H10, GL 3A1, GL 4G1, GL 12E1, GL 9F1, GL 7A12 (**DERA-CN**)

[a] Transformants assayed in each round. [b] DERA variants EP, 1E1, 2A7, 2B9 and 9F11 were used as templates of QuickChange PCR reaction respectively. [c] Residue positions targeted by site-saturation mutagenesis in round 4 were: 17, 18, 20, 21, 169, 170, 171, 173. SDM, site-directed mutagenesis; SSM, site-saturation mutagenesis.

Table S2. Amino acid substitutions compared to wild-type DERA of the representative DERA variant in each round. Amino acid substitutions that newly emerged and are retained in the final variant are highlighted in bold.

Name	Amino acid substitutions compared to wild-type DERA
EP	T18S, L20S, D22G, D24Y, C47S, I48V, F52S, K172R, T197S, P202V, A203T, R207S, G236S, S239G
9610C8	T18S, D22G, D24Y, C47S, F52S, K172L, T197S, P202V, A203T, S239G
1E1	T18S, D22G, D24Y, C47S, F52S, T197S, P202V, A203T, R207S, G236S, S239G
2A7	T18S, L20S, D22G, D24Y, C47S, F52S, T197S, P202V, A203T, R207S, G236S, S239G
2B9	T18S, L20G, D22G, D24Y, C47S, F52S, T197S, P202V, A203T, R207S, G236S, S239G
9F11	T18S, L20S, D22G, D24Y, C47S, F52S, K172R, T197S, P202V, A203T, R207S, G236S, S239G
1E1 K172L	T18S, D22G, D24Y, C47S, F52S, **K172L**, T197S, P202V, A203T, R207S, G236S, S239G
1E1 N21G/K172L	T18S, **N21G**, D22G, D24Y, C47S, F52S, **K172L**, T197S, P202V, A203T, R207S, G236S, S239G
GL 1H10	T18S, N21G, D22G, D24Y, **K37N**, C47S, F52S, K172L, T197S, P202V, A203T, R207S, G236S, S239G
GL 3A1	T18S, N21G, D22G, D24Y, **I46V**, C47S, F52S, K172L, T197S, P202V, A203T, R207S, G236S, S239G
GL 4G1	T18S, N21G, D22G, D24Y, C47S, **F52Y**, K172L, T197S, P202V, A203T, R207S, G236S, S239G
GL 12E1	T18S, N21G, D22G, D24Y, C47S, F52S, **A128T**, K172L, T197S, P202V, A203T, R207S, G236S, S239G
GL 9F1	T18S, N21G, D22G, D24Y, C47S, F52S, K172L, **P179T**, T197S, P202V, A203T, R207S, G236S, S239G
GL 7A12 (DERA-CA)	T18S, N21G, D22G, D24Y, C47S, F52S, K172L, T197S, **F200I**, P202V, A203T, R207S, G236S, S239G

Table S3. Primers used for construction of DERA-MA variants. Mutations introduced are shown in bold.

Parental DNA	Mutations		Primer Sequence (5’→ 3’)
DERA-EP	K172L	F	CCTCTACCGGT**ctg**GTGGCTGTGAACGCG
		R	GCGATGGCGAGATATTTCTGCG
DERA 1E1	K172L	F	CCTCTACCGGT**ctg**GTGGCTGTGAACGCG
		R	GCGATGGCGAGATATTTCTGCG
DERA 2A7	K172L	F	CCTCTACCGGT**ctg**GTGGCTGTGAACGCG
		R	GCGATGGCGAGATATTTCTGCG
DERA 2B9	K172L	F	CCTCTACCGGT**ctg**GTGGCTGTGAACGCG
		R	GCGATGGCGAGATATTTCTGCG
DERA 9F11	K172L	F	CCTCTACCGGT**ctg**GTGGCTGTGAACGCG
		R	GCGATGGCGAGATATTTCTGCG
DERA 1E1	Saturation K172	F	AACCTCTACCGGT**nnk**GTGGCTGTGAACGCGACGC
		R	CGTTCACAGCCAC**mnn**ACCGGTAGAGGTTTTGATG
DERA 1E1 K172L	Saturation L17	F	CTGAAATTGATGGAC**nnk**TCCACCCTGAATGGCGACTAC
		R	GCCATTCAGGGTGGA**mnn**GTCCATCAATTTCAGTGCACG
	Saturation S18	F	AAATTGATGGACCTG**nnk**ACCCTGAATGGCGACTACACC
		R	GTCGCCATTCAGGGT**mnn**CAGGTCCATCAATTTCAGTGC
		F	GGACCTGTCCACC**nnk**AATGGCGACTACACCGACG

	Saturation L20	R	TGTAGTCGCCATT**mnn**GGTGGACAGGTCCATCAAT
	Saturation G21	F	GTCCACCCTG**nnk**GGCGACTACACCGACG
		R	TGTAGTCGCC**mnn**CAGGGTGGACAGGTCC
	Saturation S169	F	GACTTCATCAAAACC**nnk**ACCGGTAAAGTGGCTGTGA
		R	CCACTTTACCGGT**mnn**GGTTTTGATGAAGTCCGCACC
	Saturation T170	F	GCGGACTTCATCAAAACCTCT**nnk**GGTAAAGTGGCTGTG
		R	CACTTTACC**mnn**AGAGGTTTTGATGAAGTCCGCACCCGC
	Saturation G171	F	ATCAAAACCTCTACC**nnk**AAAGTGGCTGTGAACGCGACG
		R	GTTCACAGCCACTTT**mnn**GGTAGAGGTTTTGATGAAGTC
	Saturation V173	F	TCAAAACCTCTACCGGTAAA**nnk**GCTGTGAACGCGACGCC
		R	GCGTTCACAGC**mnn**TTTACCGGTAGAGGTTTTGATGAAG
DERA 1E1 N21G/K172L	Randomized (error prone)	Fep	TTGTTTAACTTTAAGAAGGAGATATACATATG
		Rep	GGTGGTGGTGCTCGAG

4. Site-directed mutagenesis

The DERA variants containing the K172L mutation were obtained by site-directed mutagenesis using QuickChange PCR.[3] For the polymerase chain reaction, plasmid pET26b(+) containing the respective DERA gene, constructed as reported,[1,2] served as template. Using primer sets K172L F and R (**Table S3**), the whole length plasmid was amplified with Q5 High-Fidelity 2× Master Mix (New England Biolabs, Ipswich, MA, USA) in a 25 µL reaction volume. The following thermocycler program was used: 98 °C, 30 s (initial denaturation), followed by 20 cycles of 98 °C for 10 s, 60 °C for 30 s (annealing), 72 °C for 3 min (extension), and a final elongation step at 72 °C for 5 min. The size of the PCR product was verified using agarose gel electrophoresis. Subsequently, the verified PCR product was purified by using the PCR purification kit (QIAGEN) and digested with 0.5 µL of *Dpn*I (Thermo Fisher Scientific, Waltham, MA, USA) at 37 °C overnight to eliminate parental DNA. Subsequently, 5 µL from this mixture was used to transform an aliquot (100 µL) of chemically competent *E. coli* BL21 cells by heat shock (42 °C, 30 s) in a water bath. The cells were allowed to recover with 900 µL LB medium (37 °C, 1 h, 220 rpm). 100 µL cell culture were then spread on LB agar plates supplemented with 50 µg/mL kanamycin. After outgrowth at 37 °C overnight, 3 single colonies were picked for plasmid DNA isolation using the QIAprep Spin Miniprep Kit (QIAGEN) and for preparation of a glycerol stock. The correct clone was confirmed by DNA sequencing (Genewiz). The glycerol stock containing the correct clone was used to inoculate a cell culture for protein production.

5. Site-saturation mutagenesis

The DNA libraries were constructed by site-saturation mutagenesis using QuickChange PCR.[3] For the polymerase chain reactions, the plasmids pET26b(+) containing the appropriate DERA gene served as templates. Primers containing degenerate NNK codons that cover the 20 genetically encoded amino acids (**Table S3**) were used for the PCR reactions and each site was targeted individually. The whole length plasmid was amplified using Q5 High-Fidelity 2× Master Mix (New England Biolabs, Ipswich, MA, USA) in a 100 μL reaction volume. The following thermocycler program was used: 98 °C, 30 s (initial denaturation), followed by 18 cycles of 98 °C for 10 s, 58 °C or 60 °C (modified according to the primers Tm) for 30 s (annealing), 72 °C for 3 min (extension), and a final elongation step at 72 °C for 5 min. The size of the PCR product was verified using agarose gel electrophoresis. Subsequently, the verified PCR product was digested with 1 μL of *Dpn*I (Thermo Fisher Scientific, Waltham, MA, USA) at 37 °C overnight to eliminate parental DNA and purified by using the PCR purification kit (QIAGEN). The purified product was used to transform an aliquot (100 μL) of chemically competent *E. coli* DH5α cells by heat shock (42 °C, 30 s) in a water bath. The cells were allowed to recover with 900 μL LB medium (37 °C, 1 h, 220 rpm), then collected by centrifugation (3000 rpm, 3 min). Most of the medium was poured out and the remaining 50~200 μL was used to resuspend the cell pellet, which were then spread on LB agar plates supplemented with 50 μg/mL kanamycin. After outgrowth at 37 °C overnight, the colonies were combined for plasmid DNA isolation using the QIAprep Spin Miniprep Kit (QIAGEN) to obtain the DNA library. The library quality was assessed by sequencing the collection of plasmid DNA. The DNA library was stored at -20 °C until further use.

6. Random mutagenesis by error-prone PCR

The gene coding for DERA 1E1 N21G/K172L with a C-terminal His-tag was amplified by error-prone PCR.[4,5] The PCR reaction (100 μL) was set up with 5 ng of pET26b harbouring the gene coding for DERA 1E1 N21G/K172L, 1 U GoTaq MDxHot start polymerase (Promega Corporation, Madison, WI, USA), 0.5 μM of each primer (Fep and Rep, **Table S3**), 1 mM dCTP and dTTP, 0.2 mM dATP and dGTP, 7 mM magnesium chloride, 0.075 mM manganese chloride and GoTaq Flexbuffer. The following thermocycler program was used: 95 °C, 2 min (initial denaturation), followed by 18 cycles of each one consisted of 95 °C for 30 s (denaturation), 57 °C for 30 s (annealing), 72 °C for 1 min (extension) and a final elongation step at 72 °C for 5 min. The resulting linear epPCR fragments and the vector pET26b were digested with *Nde*I and *Xho*I restriction endonuclease (Thermo Fisher Scientific, Waltham, MA, USA), the vector backbone was dephosphorylated, the DNA was purified using a PCR purification kit (Macherey-Nagel, Düren, Germany) and ligated using T4 DNA ligase (Thermo Fisher Scientific, Waltham, MA, USA). The ligation product was purified using a PCR purification kit and used to transform electrocompetent *E. coli* DH5α cells. The cells were allowed to recover with 900 μL LB medium (37°C, 1 h, 220 rpm), then collected by centrifugation (3000 rpm, 3 min). Most of the medium was poured out and the remaining 50~200 μL was used to resuspend the cell pellet, which were then spread on LB agar plates supplemented with 50 μg/mL kanamycin. After outgrowth at 37 °C overnight, plasmids from five randomly selected colonies of each library were isolated and the DERA genes were sequenced to check the library quality. All the other colonies were resuspended together in LB medium for plasmid DNA isolation using the QIAprep Spin Miniprep Kit (QIAGEN) to obtain the DNA library. The DNA library was stored at -20 °C until further use.

7. Screening

E. coli BL21 (DE3) cell library construction

The DNA libraries were transformed into chemically competent *E. coli* BL21 (DE3) cells, and transformants were selected on LB agar plates containing 50 μg/mL kanamycin and lactose (0.2% w/v). After incubation at 37 °C for approximately 16 h, the agar plates were stored at room temperature for 1~2 h. An agar plate-based pre-screening procedure developed earlier[6] was first performed. A heated solution (~ 60 °C) of 0.6 % w/v agarose, 0.5 % v/v DMSO and 0.1 mg/mL 2-hydroxycinnamaldehyde in 10 mM sodium phosphate, pH 7.3 was poured on the agar plates. The plates were incubated for 10 min at room temperature to allow the agarose to solidify. Typically, the colonies producing active enzyme variants were stained red by the probe (2-hydroxycinnamaldehyde).[6] Stained colonies from each library were picked with sterile toothpicks, which were used to inoculate 150 μL LB medium supplemented with 50 μg/mL kanamycin and 0.2 % w/v lactose. In each plate, 2 wells were inoculated with a clean, sterile toothpick (negative control) and 5 wells (B2, D5, E7, G10, H12) were inoculated with fresh colonies producing the parental variant for activity reference. The 96-well plates were sealed with sterile gas-permeable seals (Breathe-Easy, Diversified Biotech, Boston, MA, USA) and incubated at 37 °C (180 rpm) overnight. After incubation, cell culture from 96 well plates (50 μL each well) were transferred into plates (96 well Masterblock, Greiner Bio-one, Kremsmünster, Austria) which contained 1 mL LB medium supplemented with 0.2 % w/v lactose, 0.05 % w/v glucose, and 50 μg/mL kanamycin. The 96-deep well plates were sealed with sterile gas-permeable seals and incubated at 37 °C (200 rpm) for 2 h, followed by 25 °C (200 rpm) overnight. The bacterial culture was harvested and lysed in 50 μL BugBuster (EMD Biosciences, Madison, WI, USA) containing 0.5 μL/mL benzonase. After 20 min of incubation at room temperature under vigorous shaking, the cell free extracts (CFEs) were obtained by centrifugation.

96-well plate purification

Nickle plate preparation: 100 μL Ni-sepharose was loaded into each well of AcroPrep™ 96-well filter plates, 1 mL (Pall Life Science). The nickle plate was washed with 1.6 mL demi-water and equilibrated with 1.6 mL 20 mM potassium phosphate, 30 mM imidazole, pH 7 per well.

Protein bind, wash and elute: The cell free extracts (CFEs) in 96 well Masterblock were loaded into the nickle plate. The nickle plate was washed with 600 μL 20 mM potassium phosphate, 30 mM imidazole, pH 7 per well. The retained wash buffer was removed by centrifuge 800 x g for 1 min. The nickle plate was placed on a 96-well microplate (F-bottom, 300 uL, Greiner Bio-one, Kremsmünster, Austria), and the retained protein was eluted with 150 μL 20 mM potassium phosphate, 300 mM imidazole, pH 7 by centrifugation at 800 g for 2 min.

For buffer exchange, the PD MultiTrap™ G-25 plate was placed on the 96-well microplate (F-bottom, 300 uL, Greiner Bio-one, Kremsmünster, Austria). The PD MultiTrap™ G-25 plate was equilibrated with 5 times with 300 μL 20 mM potassium phosphate, pH 7 per well. Subsequently, 130 μL elute protein solution was slowly added to the middle of the packed bed, followed by centrifuge 800 g for 2 min, to collect the proteins.

Screening

For the screening, 111 μL of the pure protein was transferred to a 96-well microplate (UV-star F-bottom microplate, Greiner Bio-one, Kremsmünster, Austria). 20 mM potassium phosphate, pH 7, buffer containing the substrate mixture was then added to each well of the plate to initiate the activity screening. The final reaction mixture (120 μL volume) consisted of the following: 111 μL pure protein, 0.2 mM **1a**, 4 mM **2**, 7.5 % (v/v) EtOH and 20 mM potassium phosphate, pH 7. The reaction rate was measured by monitoring the cinnamaldehyde depletion (at 290 nm) at 25 °C in a plate reader (SPECTROstar Omega). The variants with the highest increase in activity compared to the reference were picked from the 96-well plate for plasmid isolation, DNA sequencing and further mutagenesis.

8. Enzyme expression and purification

For heterologous expression of all enzymes, *E. coli* BL21(DE3) (New England BioLabs, NEB) was used as host organism. Transformations were performed by heat shock at 42 °C for 30 seconds.

Expression and purification of DERA

All genes encoding DERA enzymes with a C-terminal His-tag were cloned in the pET26b vector. A single colony (or glycerol stock) was picked to inoculate 5 mL pre-culture (LB medium with 50 μg/mL kanamycin). Typically, following incubation at 37 °C and 200 rpm for 12-16 h, 1 mL pre-culture was used to inoculate 100 mL TB medium with 50 μg/mL kanamycin. The cells were allowed to grow at 37 °C until an OD_{600} of 0.6~1 was reached. Expression of the enzymes was performed by adding 0.1 mM of isopropyl β-D-1-thiogalactopyranoside (IPTG) and cultures were grown at 25 °C (200 rpm) overnight. The next day, the cells were harvested by centrifugation (3.700 rpm, 40 min, 4°C), resuspended in 20 mM KPi, 15 mM imidazole, pH 7, and lysed by ultrasonication, followed by centrifugation to obtain the cell free extract (CFE).

Protein purification was performed by Ni-NTA affinity chromatography using Ni-NTA HisTrap™ HP chromatography columns (Cytiva) containing 3 mL Ni-sepharose. The CFE was loaded into the column, which has been previously equilibrated with 20 mM KPi, 30 mM imidazole, pH 7. After loading of the filtered lysate, the column was washed with 30 mL 20 mM KPi, 30 mM imidazole, pH 7. The retained protein was eluted with 20 mM KPi, 300 mM imidazole, pH 6.5. Eluted fractions were used for buffer exchange using PD-10 Sephadex™ G-25M columns (Cytiva) and enzymes were obtained in 20 mM potassium phosphate buffer, pH 7. The protein concentration was determined by absorbance at 280 nm measured with a Nanodrop 2000 (Thermo Scientific) with the theoretical molecular mass and extinction coefficient estimated using the Expasy webserver (https://web.expasy.org/protparam/). Typically, a protein yield of 30-60 mg per gram of cell culture was obtained. The purity of the enzymes was examined by SDS-PAGE analysis (**Figure S2**). The remaining protein solution was snap-frozen in liquid nitrogen and stored at -20 °C until further use.

Expression and purification of *At*HNL

*At*HNL was cloned in the pET21a vector with a C-terminus His-tag. A single colony (or glycerol stock) was picked to inoculate 5 mL pre-culture (LB medium with 50 μg/mL ampicillin). Following incubation at 37 °C and 200 rpm for 12-16 h, 5 mL pre-culture was used to inoculate 500 mL TB medium with 100 μg/mL ampicillin. The cells were allowed to grow at 37 ˚C until an OD_{600} of 0.6~1 was reached. Expression of the enzyme was performed by adding 0.2 mM of isopropyl β-D-1-thiogalactopyranoside (IPTG) and cultures were grown at 16 °C (200 rpm) overnight. The next day, the cells were harvested by centrifugation (3700 rpm, 40 min, 4 °C), resuspended in 20 mM KPi, 15 mM imidazole, pH 7, and lysed by ultrasonication, followed by centrifugation to obtain the cell free extract (CFE).

Protein purification was performed by Ni-NTA affinity chromatography using Ni-NTA HisTrap™ HP chromatography columns (Cytiva) containing 5 mL Ni-sepharose. The CFE was loaded into the column, which has been previously equilibrated with 20 mM KPi, 30 mM imidazole, pH 7. After loading of the filtered lysate, the column was washed with 30 mL 20 mM KPi, 50 mM imidazole, pH 7. The retained protein was eluted with 20 mM KPi, 500 mM imidazole, pH 7. Eluted fractions were used for buffer exchange using PD-10 Sephadex™ G-25M columns (Cytiva) and enzymes were obtained in 20 mM potassium phosphate buffer, pH 7. The protein concentration was determined by absorbance at 280 nm measured with a Nanodrop 2000 (Thermo Scientific) with the theoretical molecular mass and extinction coefficient estimated using the Expasy webserver (https://web.expasy.org/protparam/). A protein yield of 20-40 mg per gram of cell culture was obtained. The purity of the enzymes was examined by SDS-PAGE analysis (**Figure S2**). The remaining protein solution was snap-frozen in liquid nitrogen and stored at -20 °C until further use.

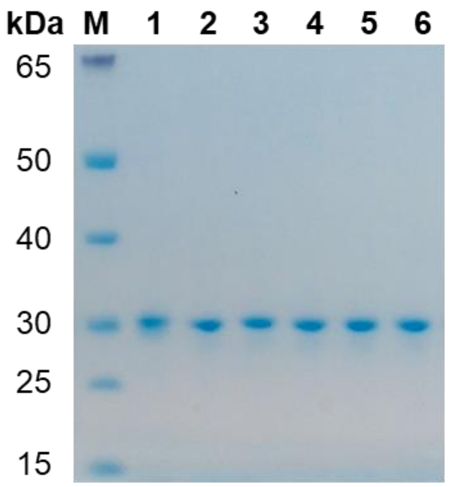

Figure S2. SDS-PAGE analysis (4-12% NuPAGE Bis-Tris) of purified DERA variants and *At*HNL. Lane M: PageRuler^{TM} Prestained Protein ladder, Lane 1: *At*HNL, Lane 2: DERA wildtype, Lane 3: DERA 1E1, Lane 4: DERA 1E1 L172L, Lane 5: DERA 1E1 N21G/K172L, Lane 6: DERA-CN.

9. Enzyme activity assay

The DERA cyanation activity assays were performed at room temperature. Fresh stock solutions of **1a** (40 mM in EtOH) and **2** (400 mM) were prepared. The reaction mixture (400 µL) consisted of **1a** (1 mM), **2** (20 mM), and DERA variant (50 µM) in 20 mM KPi (pH 6.5) with 7.5% (v/v) EtOH. For each enzymatic reaction, a negative control reaction without enzyme (enzyme replaced by buffer) was set up in parallel. After 18 h, the reaction mixture was used for extraction with EtOAc. Calibration curves of compound **1a** were obtained using 1 mM mesitylene as the internal standard (**Figure S16, S17**). The GC-FID data for the analytical scale reactions were fit in the equation to determine the conversion. The enantiomeric ratio (e.r.) and diastereomeric ratio (d.r.) were determined by chiral normal phase HPLC analysis (**Figures S18**).

10. Upscale synthesis of **4a** catalyzed by DERA-EP

A reaction mixture was set up containing 2 mM **1a**, 20 mM **2**, 7.5% (v/v) EtOH and 300 μM of DERA-EP in a final volume of 280 mL in 20 mM HEPES buffer (pH 6.5). A negative control reaction without enzyme (replaced by buffer) was set up in parallel. The reaction mixture was incubated in a 500 mL Erlenmeyer flask at room temperature, started by adding 2 mM **1a** and 20 mM **2**. After 60 h, the reaction mixture was extracted three times with 200 mL ethyl acetate. The organic layers were combined and evaporated until 30 mL was left, followed by washing with 30 mL brine, followed by filtration using hydrophobic filter paper (MACHEREY-NAGEL, phase separation) and concentrated *in vacuo*. The resulting crude product was analyzed by HRMS (**Figure S3**) and NMR (**Figure S5**) to determine the product structure.

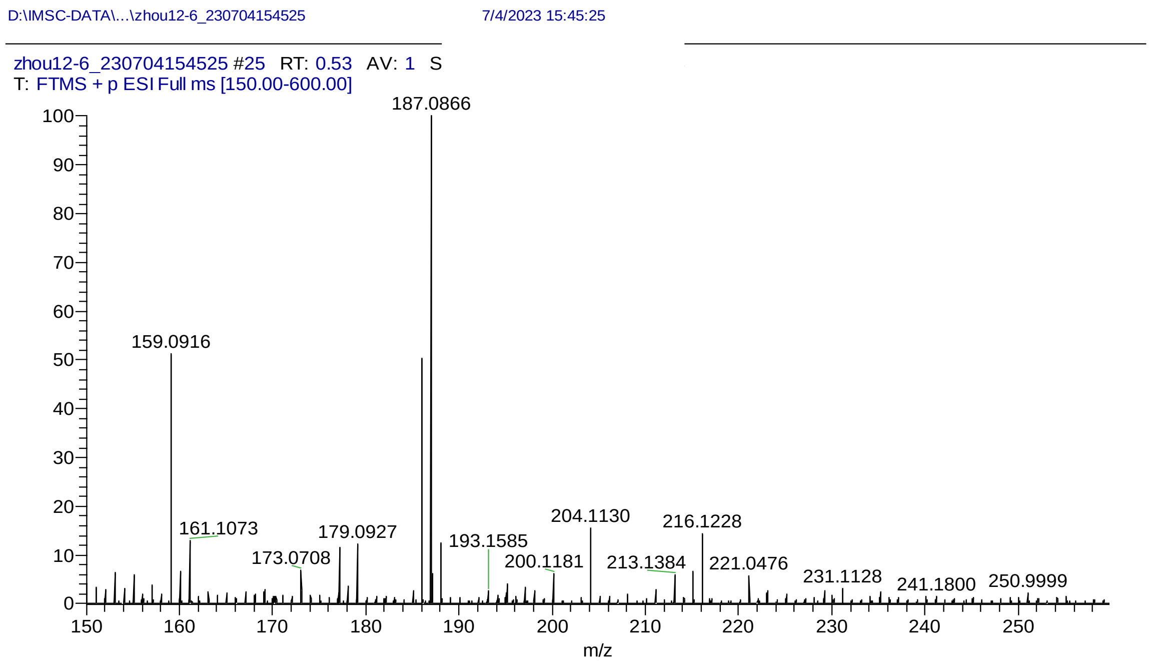

Figure S3. ESI-MS of crude 2-hydroxy-4-phenylpentanedinitrile (**4a**) obtained by upscale synthesis using DERA-EP. HRMS–ESI$^+$ (*m/z*): [M + H]$^+$ calcd for $C_{11}H_{11}N_2O$, 187.0793; found, 187.0866.

11. Buffer optimization

Table S4. Buffer optimization for the cascade reaction.[a]

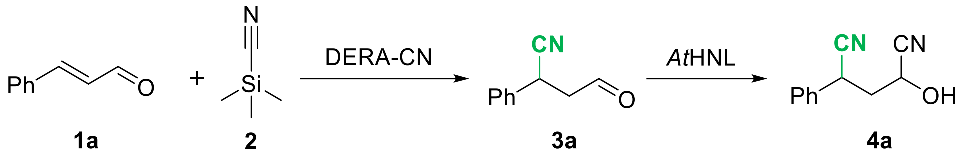

Entry	Buffer	Conv. (%)	e.r.[b]	d.r.[c]
1	20 mM KPi, pH 7	55	85:15	60:40
2	50 mM citrate pH 2,7	0	n.d.	n.d.
3	50 mM citrate pH 3,5	0	n.d.	n.d.
4	50 mM citrate pH 4,5	0	n.d.	n.d.
5	50 mM citrate pH 5,5	66	99:1	59:41
6	20 mM HEPES, 100 mM NaCl, pH 8,3	26	81:19	59:41
7	20 mM HEPES, 100 mM NaCl, pH 10,6	30	74:26	58:42

[a] Analytical scale reactions were performed with 1 mM **1a**, 20 mM **2**, 20 µM DERA-CN and 10 µM *At*HNL, 7.5% v/v EtOH at room temperature, reaction volume = 400 µL, reaction time = 24 h. [b] The enantiomeric ratio was determined by chiral normal phase HPLC. [c] The diastereomeric ratio was determined by chiral normal phase HPLC. n.d.: not determined.

Table S5. Buffer optimization of DERA-CN catalyzed cyanation.[a]

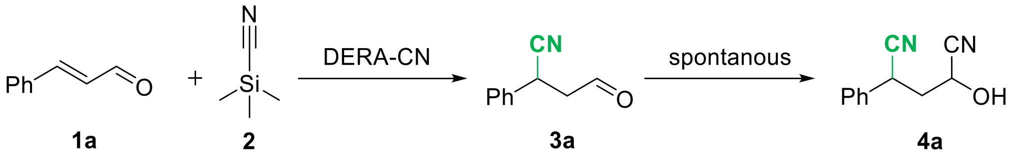

Entry	Buffer	Conv. (%)	e.r.[b]	d.r.[c]
1	20 mM KPi, pH7	56	61:39	58:42
2	50 mM citrate pH 5,5	45	99:1	55:45

[a] Analytical scale reactions were performed with 1 mM **1a**, 20 mM **2** and 50 µM DERA-CN, 7.5% v/v EtOH at room temperature, reaction volume = 400 µL, reaction time = 18 h, Enzymatic product **3a** undergoes spontaneous cyanation to form compound **4a**, which results in a poor diastereomeric ratio of **4a**. [b] The enantiomeric ratio was determined by chiral normal phase HPLC. [c] The diastereomeric ratio was determined by chiral normal phase HPLC.

Table S6. Control experiment.[a]

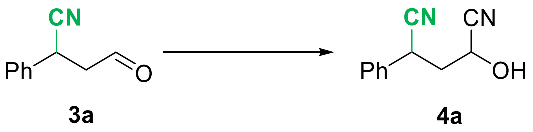

Entry	Buffer	Conv. (%)	e.r.[b]	d.r.[c]
1	20 mM KPi, pH7	84	54:46	55:45
2	50 mM citrate pH 5,5	>99	53:47	55:45

[a] Analytical scale reactions were performed with 1 mM **3a** (chemically synthesized) and 20 mM **2**, 7.5% v/v EtOH at room temperature, reaction volume = 400 µL, reaction time = 18 h. [b] The enantiomeric ratio was determined by chiral normal phase HPLC. [c] The diastereomeric ratio was determined by chiral normal phase HPLC.

12. Substrate scope analysis of DERA-CN

All reactions were performed at room temperature. The reaction mixture (400 µL) consisted of substrate **1** (1 mM), **2** (20 mM), and purified DERA-CN (50 µM) in 50 mM citrate (pH 5.5) with 7.5% (v/v) EtOH (ACN for substrate **1d** and **1g**) as cosolvent. For each enzymatic reaction, a negative control reaction without enzyme (enzyme replaced by buffer) was set up in parallel. After a reaction time of 24 h, the reaction mixture was used for extraction with EtOAc. Calibration curves of compound **1a**, **1f**, **1g** were obtained using 1 mM mesitylene as the internal standard (**Figure S16, S17**). The GC-FID data for the analytical scale reactions were fit in the equation to determine the conversion. The enantiomeric ratio (e.r.) and diastereomeric ratio (d.r.) were determined by chiral normal phase HPLC analysis (**Figures S19-S27**).

13. Synthesis of the racemic reference compounds **4**

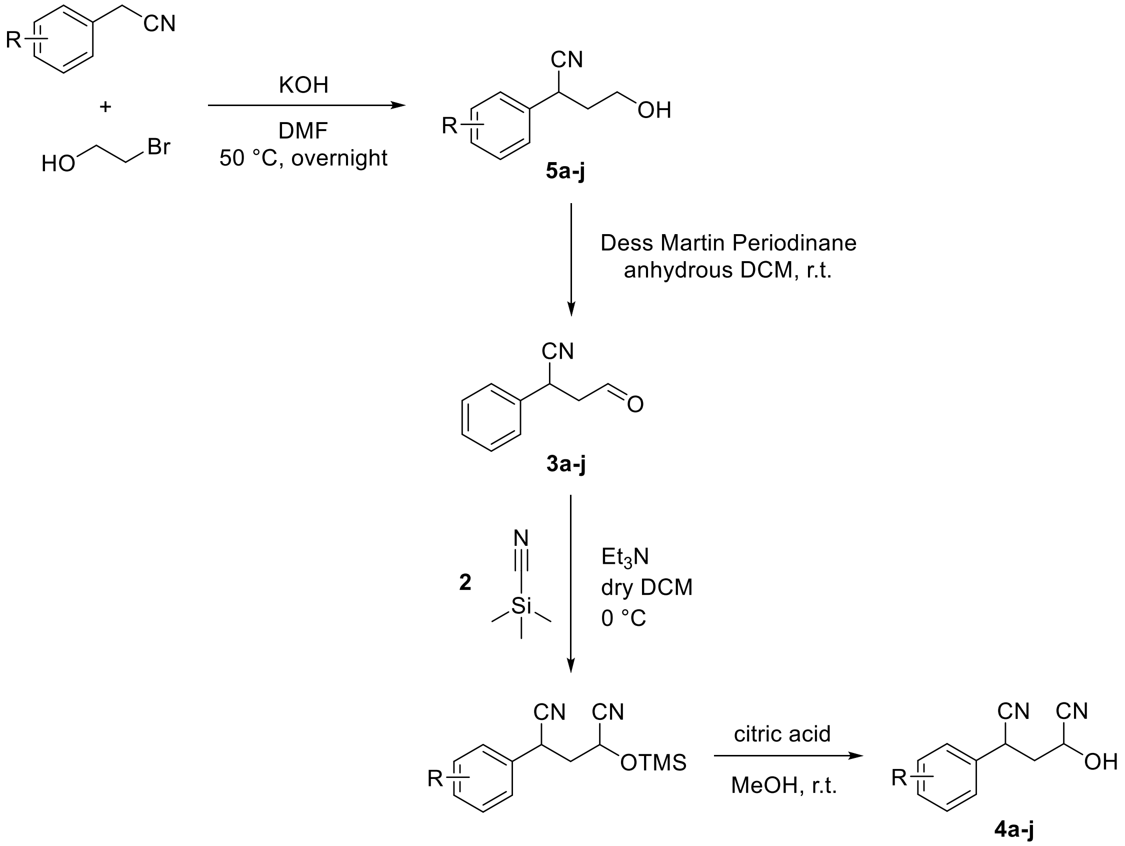

The synthesis of racemic reference compounds was performed according to a modified previously published procedure[7–10].Typically, to a suspension of KOH (2.3 g, 4.6 equiv) in 6.8 mL DMF, 9 mmol benzyl cyanide derivatives were added. Then 9 mmol 2-bromoethanol were added dropwise, during which the temperature may not exceed 50 °C. After the addition was complete, the mixture was stirred overnight at 50 °C. The mixture was poured on ice and extracted 3 times with ethyl acetate. The combined organic phases were filtrated and concentrated *in vacuo*. Flash purification (**Table S7**) gave racemic compound **5a-j**.

Table S7. Flash purification conditions of racemic reference compound **5a-j**.

Entry	1	R	Eluent
1	**a**	H	35% EtOAc in pentane
2	**b**	*p*-F	40% EtOAc in pentane
3	**c**	*p*-Cl	45% EtOAc in pentane
4	**d**	*p*-Br	45% EtOAc in pentane
5	**e**	*o*-F	35% EtOAc in pentane
6	**f**	*m*-CF_3	40% EtOAc in pentane
7	**g**	*p*-OMe	45% EtOAc in pentane
8	**h**	*p*-Me	30% EtOAc in pentane
9	**i**	*o*-OMe	45% EtOAc in pentane
10	**j**	*m*-Me	30% EtOAc in pentane

Dess Martin periodinane (1 g, 1.2 equiv) was added to a solution of racemic compound **5a-j** (2 mmol) in anhydrous dicholoromethane (20 mL). After stirring overnight at room temperature, the reaction mixture was diluted with diethyl ether and poured into saturated aqueous $NaHCO_3$ containing a 7-fold excess of $Na_2S_2O_3$. The mixture was stirred to dissolve the solid and the layers were separated. The ether layer is extracted with saturated $NaHCO_3$ followed by filtration and concentration in vacuo.

The crude mixture was dissolved in dry dicholoromethane to get 0.25 M final concentration, then 1 eq of trimethylsilyl cyanide **2** and a catalytic amount (10 mol%) of triethylamine were added at 0 °C. The mixture was stirred overnight at the same temperature and the solvent was removed under reduced pressure.

The crude mixture was dissolved in MeOH to get 0.1 M final concentration, then a catalytic amount (10 mol%) of citric acid was added. The reaction mixture was stirred overnight at room temperature. Afterwards, the mixture was extracted 3 times with ethyl acetate, filtrated and concentrated in vacuo. Flash purification (**Table S8**) gave racemic compound **4a-j.**

Table S8. Flash purification conditions of racemic reference compound **4a-j**.

Entry	1	R	Eluent
1	**a**	H	40% MTBE in pentane
2	**b**	*p*-F	35% EtOAc in pentane
3	**c**	*p*-Cl	25% EtOAc in pentane
4	**d**	*p*-Br	25% EtOAc in pentane
5	**e**	*o*-F	20% EtOAc in pentane
6	**f**	*m*-CF_3	30% EtOAc in pentane
7	**g**	*p*-OMe	40% EtOAc in pentane
8	**h**	*p*-Me	30% MTBE in pentane
9	**i**	*o*-OMe	28% EtOAc in pentane
10	**j**	*m*-Me	20% EtOAc in pentane

***Rac*-2-hydroxy-4-phenylpentanedinitrile (4a)**

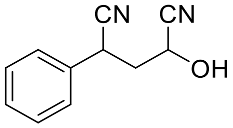

^{1}H NMR (600 MHz, $CDCl_3$, mixture of diastereomers, d.r. = 55:45) δ 7.43 – 7.35 (m, 5H), 4.74 – 4.68 (m, 0.55H), 4.32 (dd, *J* = 9.3, 5.0 Hz, 0.45H)*, 4.16 (dd, *J* = 9.3, 6.5 Hz, 0.57H), 4.05 (dd, *J* = 9.1, 6.6 Hz, 0.47H)*, 2.60 (ddd, *J* = 13.9, 9.3, 6.6 Hz, 0.51H)*, 2.40 (m, 1.02H), 2.33 (ddd, *J* = 14.1, 9.1, 5.0 Hz, 0.56H)*. * minor diastereomer.

***Rac*-2-(4-fluorophenyl)-4-hydroxypentanedinitrile (4b)**

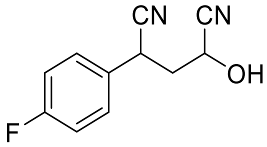

^{1}H NMR (600 MHz, $CDCl_3$, mixture of diastereomers, d.r. = 62:38) δ 7.35 (ddt, *J* = 7.1, 4.8, 2.1 Hz, 2H), 7.12 (td, *J* = 8.6, 2.7 Hz, 2H), 4.73 (t, *J* = 7.0 Hz, 0.62H), 4.34 (d, *J* = 8.1 Hz, 0.38H)*, 4.17 (dd, *J* = 8.8, 7.0 Hz, 0.56H), 4.07 (dd, *J* = 8.9, 6.8 Hz, 0.37H)*, 3.31 (s, 0.57H), 3.06 (d, *J* = 5.2 Hz, 0.37H)*, 2.60 (ddd, *J* = 13.9, 9.1, 6.8 Hz, 0.39H)*, 2.38 (dd, *J* = 8.6, 6.3 Hz, 1.2H), 2.31 (ddd, *J* = 14.0, 8.9, 5.1 Hz, 0.36H)*. * minor diastereomer. HRMS–ESI$^+$ (*m/z*): [M + NH_4]$^+$ calcd for $C_{11}H_{13}FN_3O$, 220.1037; found, 222.1030.

***Rac*-2-(4-chlorophenyl)-4-hydroxypentanedinitrile (4c)**

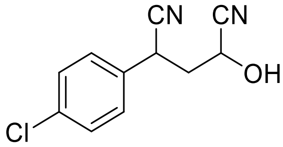

^{1}H NMR (600 MHz, $CDCl_3$, mixture of diastereomers, d.r. = 72:28) δ 7.45 – 7.35 (m, 2H), 7.31 (dd, *J* = 8.6, 2.4 Hz, 2H), 4.76 – 4.71 (m, 0.72H), 4.35 (dd, *J* = 9.1, 5.1 Hz, 0.28H)*, 4.19 – 4.15 (m, 0.72H), 4.06 (dd, *J* = 8.8, 6.8 Hz, 0.25H)*, 3.20 (s, 0.69H), 2.94 (s, 0.25H)*, 2.60 (ddd, *J* = 14.1, 9.1, 6.8 Hz, 0.26H)*, 2.42 – 2.35 (m, 1.45H), 2.31 (ddd, *J* = 14.0, 8.8, 5.1 Hz, 0.26H)*. * minor diastereomer. HRMS–ESI$^+$ (*m/z*): [M + NH_4]$^+$ calcd for $C_{11}H_{13}ClN_3O$, 238.0742; found, 238.0735.

***Rac*-2-(4-bromophenyl)-4-hydroxypentanedinitrile (4d)**

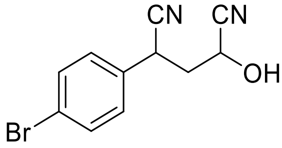

^{1}H NMR (600 MHz, $CDCl_3$, mixture of diastereomers, d.r. = 62:37) δ 7.61 – 7.52 (m, 2H), 7.26 – 7.24 (m, 2H), 4.77 – 4.69 (m, 0.62H), 4.35 (dd, *J* = 9.1, 5.1 Hz, 0.37H)*, 4.18 – 4.14 (m, 0.68H), 4.05 (dd, *J* = 8.8, 6.8 Hz, 0.38H)*, 2.59 (ddd, *J* = 14.1, 9.1, 6.8 Hz, 0.38H)*, 2.43 – 2.34 (m, 1.29H), 2.31 (ddd, *J* = 14.0, 8.8, 5.1 Hz, 0.39H)*. * minor diastereomer. HRMS–ESI$^+$ (*m/z*): [M + NH_4]$^+$ calcd for $C_{11}H_{13}BrN_3O$, 282.0237; found, 282.0233.

***Rac*-2-(2-fluorophenyl)-4-hydroxypentanedinitrile (4e)**

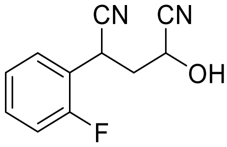

^{1}H NMR (600 MHz, $CDCl_3$, mixture of diastereomers, d.r. = 53:47) δ 7.50 – 7.34 (m, 2H), 7.26 - 7.19 (m, 1H), 7.18 -7.09 (m, 1H), 4.75 – 4.69 (m, 0.53H), 4.43 (dd, *J* = 9.2, 6.3 Hz, 0.50H), 4.40 – 4.36 (m, 0.47H)*, 4.32 (q, *J* = 8.4, 7.7 Hz, 0.40H)*, 2.61 – 2.55 (m, 0.44H)*, 2.46 – 2.37 (m, 1.40H). * minor diastereomer. HRMS–ESI$^+$ (*m/z*): [M + NH_4]$^+$ calcd for $C_{11}H_{13}FN_3O$, 220.1037; found, 222.1033.

Rac-2-hydroxy-4-(3-(trifluoromethyl)phenyl)pentanedinitrile (4f)

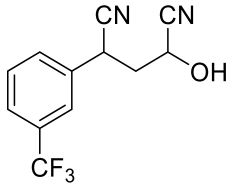

^{1}H NMR (600 MHz, $CDCl_3$, mixture of diastereomers, d.r. = 60:40) δ 7.70 – 7.56 (m, 4H), 4.79 (dd, *J* = 7.8, 6.0 Hz, 0.60H), 4.38 (dd, *J* = 9.0, 5.3 Hz, 0.41H)*, 4.27 (dd, *J* = 8.9, 7.0 Hz, 0.63H), 4.16 (dd, *J* = 8.6, 7.1 Hz, 0.35H)*, 2.64 (ddd, *J* = 14.1, 9.0, 7.1 Hz, 0.39H)*, 2.43 (dd, *J* = 8.8, 6.1 Hz, 1.28H), 2.36 (ddd, *J* = 14.0, 8.6, 5.3 Hz, 0.36H)*. * minor diastereomer. HRMS–ESI$^+$ (*m/z*): $[M + NH_4]^+$ calcd for $C_{12}H_{13}F_3N_3O$, 272.1005; found, 272.1003.

Rac-2-hydroxy-4-(4-methoxyphenyl)pentanedinitrile (4g)

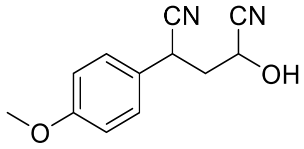

^{1}H NMR (600 MHz, $CDCl_3$, mixture of diastereomers, d.r. = 60:40) δ 7.29 – 7.25 (m, 2H), 6.95 – 6.91 (m, 2H), 4.69 (dt, *J* = 8.3, 5.1 Hz, 0.60H), 4.32 (dt, *J* = 9.6, 5.0 Hz, 0.40H)*, 4.16 – 4.09 (m, 1.00H), 4.01 (dd, *J* = 9.3, 6.4 Hz, 0.37H)*, 3.82 (d, *J* = 1.2 Hz, 2.86H), 3.21 (d, *J* = 4.9 Hz, 0.55H), 2.93 (d, *J* = 5.6 Hz, 0.34H)*, 2.58 (ddd, *J* = 14.1, 9.4, 6.5 Hz, 0.36H)*, 2.41 – 2.33 (m, 1.18H), 2.30 (ddd, *J* = 14.1, 9.4, 4.8 Hz, 0.34H)*. * minor diastereomer. HRMS–ESI$^+$ (*m/z*): $[M + H]^+$ calcd for $C_{12}H_{13}N_2O_2$, 217.0972; found, 217.0965.

Rac-2-hydroxy-4-(_p_-tolyl)pentanedinitrile (4h)

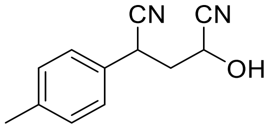

^{1}H NMR (600 MHz, $CDCl_3$, mixture of diastereomers, d.r. = 53:47) δ 7.25 – 7.20 (m, 4H), 4.74 – 4.67 (m, 0.53H), 4.33 (dt, *J* = 9.4, 5.3 Hz, 0.46H)*, 4.16 – 4.11 (m, 0.61H), 4.02 (dd, *J* = 9.3, 6.5 Hz, 0.50H)*, 3.08 (d, *J* = 5.4 Hz, 0.63H), 2.78 (d, *J* = 5.8 Hz, 0.48H)*, 2.59 (ddd, *J* = 14.0, 9.3, 6.5 Hz, 0.50H)*, 2.40 – 2.36 (m, 4.53H), 2.32 (ddd, *J* = 14.0, 9.2, 4.8 Hz, 0.54H)*. * minor diastereomer. HRMS–ESI$^+$ (*m/z*): $[M + NH_4]^+$ calcd for $C_{12}H_{16}N_3O$, 218.1288; found, 218.1281.

Rac-2-hydroxy-4-(2-methoxyphenyl)pentanedinitrile (4i)

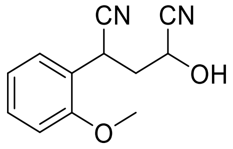

^{1}H NMR (600 MHz, $CDCl_3$, mixture of diastereomers, d.r. = 50:50) δ 7.42 – 7.32 (m, 2H), 7.04 -6.98 (m, 1H), 6.96 – 6.91 (m, 1H), 4.73 – 4.65 (m, 0.50H) *, 4.48 (dd, *J* = 8.4, 6.7 Hz, 0.50H)*, 4.40 (dd, *J* = 8.8, 5.4 Hz, 0.86H), 4.34 (dd, *J* = 8.0, 7.0 Hz, 0.86H), 3.90 (d, *J* = 7.5 Hz, 4.19H), 2.52 (ddd, *J* = 13.9, 8.8, 7.0 Hz, 0.92H)*, 2.47 – 2.37 (m, 1.92H). * minor diastereomer. HRMS–ESI$^+$ (*m/z*): $[M + NH_4]^+$ calcd for $C_{12}H_{16}N_3O_2$, 234.1237; found, 234.1232.

Rac-2-hydroxy-4-(_m_-tolyl)pentanedinitrile (4j)

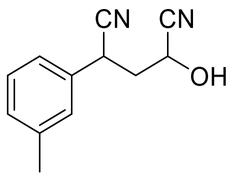

^{1}H NMR (600 MHz, $CDCl_3$, mixture of diastereomers, d.r. = 78:22) δ 7.30 (t, *J* = 7.6 Hz, 1H), 7.21 – 7.13 (m, 3H), 4.74 – 4.69 (m, 0.78H), 4.34 (dd, *J* = 9.3, 4.9 Hz, 0.22H)*, 4.16 – 4.10 (m, 0.91H), 4.02 (dd, *J* = 9.1, 6.6 Hz, 0.23H)*, 2.58 (dd, *J* = 9.3, 6.6 Hz, 0.13H)*, 2.38 (m, 4.67H), 2.33 (td, *J* = 9.1, 4.5 Hz, 0.20H)*. * minor diastereomer.

14. DNA and Protein sequences

> DNA sequence of DERA-CN 5'→3'

ATGACTGATCTGAAAGCAAGCAGCCTGCGTGCACTGAAATTGATGGACCTGTCCACCCTGGGGGGCGACTACACC
GACGAGAAAGTAATTGCTCTGTGTCATCAGGCCAAAACCCCGGTCGGCAATACCGCCGCTATCAGTATCTATCCT
CGCTCTATCCCGATTGCTCGCAAAACACTGAAAGAGCAGGGCACCCCGGAAATCCGTATTGCTACGGTAACCAAC
TTCCCACACGGTAACGACGACATCGAAATCGCGCTGGCAGAAACCCGTGCGGCAATCGCCTACGGAGCCGATGAA
GTTGACGTGGTGTTCCCGTACCGCGCGCTGATGGCGGGTAACGAGCAGGTTGGTTTTGACCTGGTGAAAGCTTGT
AAAGAGGCCTGCGCGGCAGCGAATGTACTGCTGAAAGTGATCATCGAAACCGGCGAACTGAAAGACGAAGCGCTG
ATCCGTAAAGCGTCTGAAATCTCCATCAAAGCGGGTGCGGACTTCATCAAAACCTCTACCGGTCTGGTGGCTGTG
AACGCGACGCCGGAAAGCGCGCGCATCATGATGGAAGTGATCCGTGATATGGGCGTAGAAAAATCCGTTGGTATC
AAAGTGACGGGCGGCGTGAGTACTGCGGAAGATGCGCAGAAATATCTCGCCATCGCAGATGAGCTGTTCGGTGCT
GACTGGGCAGATGCGCGTCACTACCGCTTTAGTGCTTCCGGCCTGCTGGCAAGCCTGTTGAAAGCGCTGGGCCAC
GGTGATGGTAAGAGCGCCAGCAGCTACCTCGAGCACCACCACCACCACCACTGA

> protein sequence of DERA-CN

MTDLKASSLRALKLMDLSTLGGDYTDEKVIALCHQAKTPVGNTAAISIYPRSIPIARKTLKEQGTPEIRIATVTN
FPHGNDDIEIALAETRAAIAYGADEVDVVFPYRALMAGNEQVGFDLVKACKEACAAANVLLKVIIETGELKDEAL
IRKASEISIKAGADFIKTSTGLVAVNATPESARIMMEVIRDMGVEKSVGIKVTGGVSTAEDAQKYLAIADELFGA
DWADARHYRFSASGLLASLLKALGHGDGKSASSYLEHHHHHH

> DNA sequence of *At*HNL 5'→3'

ATGGAACGCAAACATCATTTTGTGCTGGTGCATAACGCGTATCATGGCGCGTGGATTTGGTATAAACTGAAACCG
CTGCTGGAAAGCGCGGGCCATCGCGTGACCGCGGTGGAACTGGCGGCGAGCGGCATTGATCCGCGCCCGATTCAA
GCGGTGGAAACCGTGGATGAATATAGCAAACCGCTGATTGAAACCCTGAAAAGCCTGCCGGAAAACGAAGAAGTG
ATTCTGGTGGGCTTTAGCTTTGGCGGCATTAACATTGCGCTGGCGGCGGATATTTTTCCGGCGAAAATTAAAGTG
CTGGTGTTTCTGAACGCGTTTCTGCCGGATACCACCCATGTGCCGAGCCATGTGCTGGATAAATATATGGAAATG
CCGGGCGGCCTGGGCGATTGCGAATTTAGCAGCCATGAAACCCGCAACGGCACCATGAGCCTGCTGAAAATGGGC
CCGAAATTTATGAAAGCGCGCCTGTATCAGAACTGCCCGATTGAAGATTATGAACTGGCGAAAATGCTGCATCGC
CAAGGCAGCTTTTTTACCGAAGATCTGAGCAAAAAAGAAAAATTTAGCGAAGAAGGCTATGGCAGCGTGCAGCGC
GTGTATGTGATGAGCAGCGAAGATAAAGCGATTCCGTGCGATTTTATTCGCTGGATGATTGATAACTTTAACGTG
AGCAAAGTGTATGAAATTGATGGCGGCGATCACATGGTGATGCTGAGCAAACCGCAGAAACTGTTTGATAGCCTG
AGCGCGATTGCGACCGATTATATGCTCGAGCACCACCACCACCACCACTGA

> protein sequence of *At*HNL

MERKHHFVLVHNAYHGAWIWYKLKPLLESAGHRVTAVELAASGIDPRPIQAVETVDEYSKPLIETLKSLPENEEV
ILVGFSFGGINIALAADIFPAKIKVLVFLNAFLPDTTHVPSHVLDKYMEMPGGLGDCEFSSHETRNGTMSLLKMG
PKFMKARLYQNCPIEDYELAKMLHRQGSFFTEDLSKKEKFSEEGYGSVQRVYVMSSEDKAIPCDFIRWMIDNFNV
SKVYEIDGGDHMVMLSKPQKLFDSLSAIATDYMLEHHHHHH

Figure S4. DNA and protein sequence of DERA-CN and *At*HNL, the mutated amino acids are shown in red.

15. NMR spectra

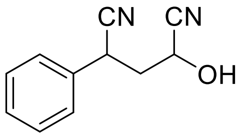

enzymatic

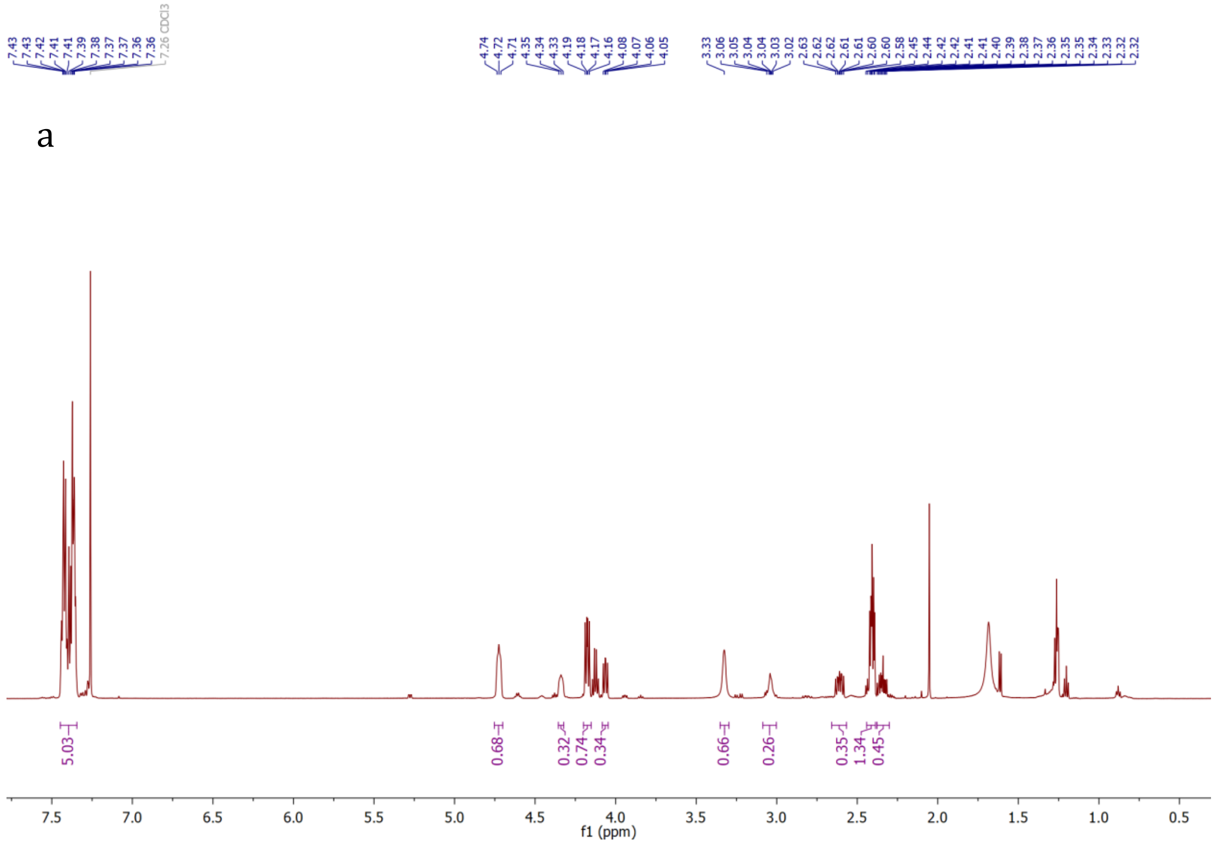

b

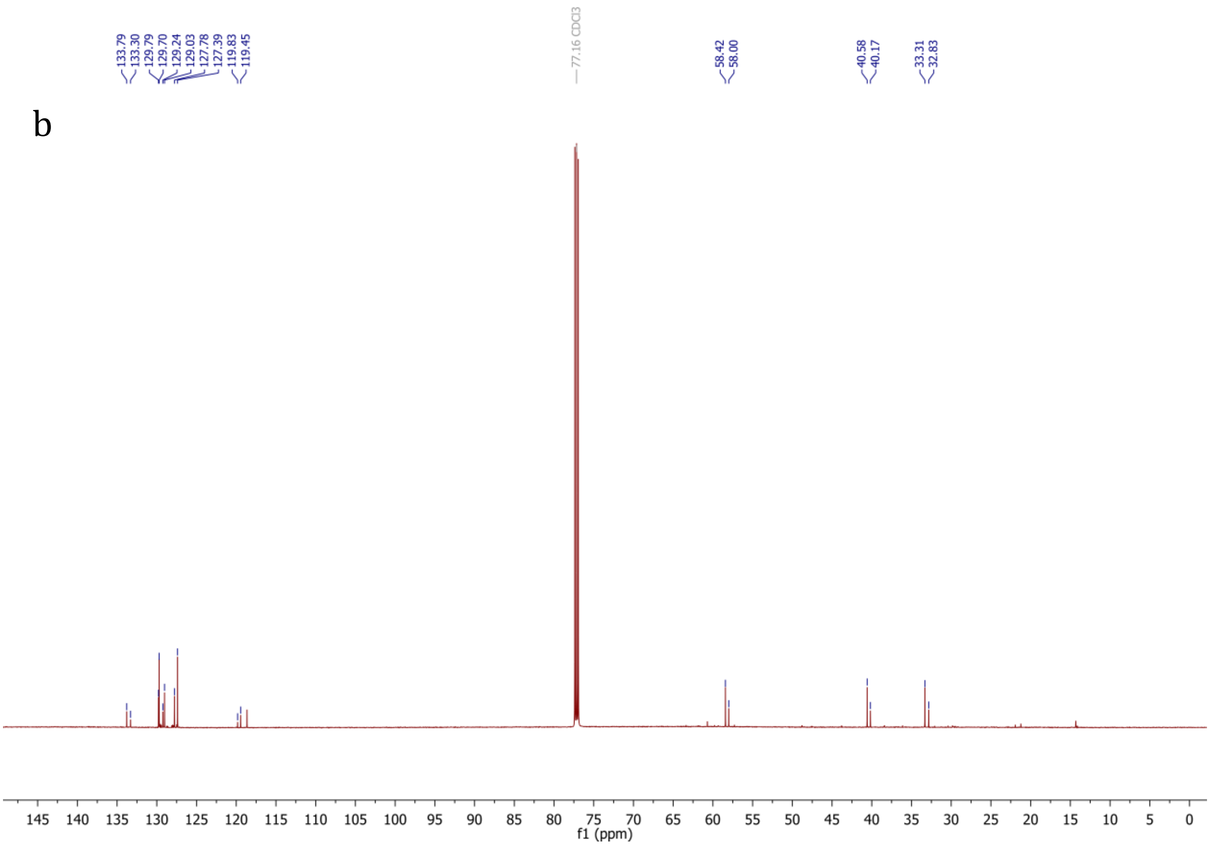
133.79
133.30
129.79
129.70
129.24
129.03
127.78
127.39
119.83
119.45
77.16 CDCl3
58.42
58.00
40.58
40.17
33.31
32.83
f1 (ppm)

c

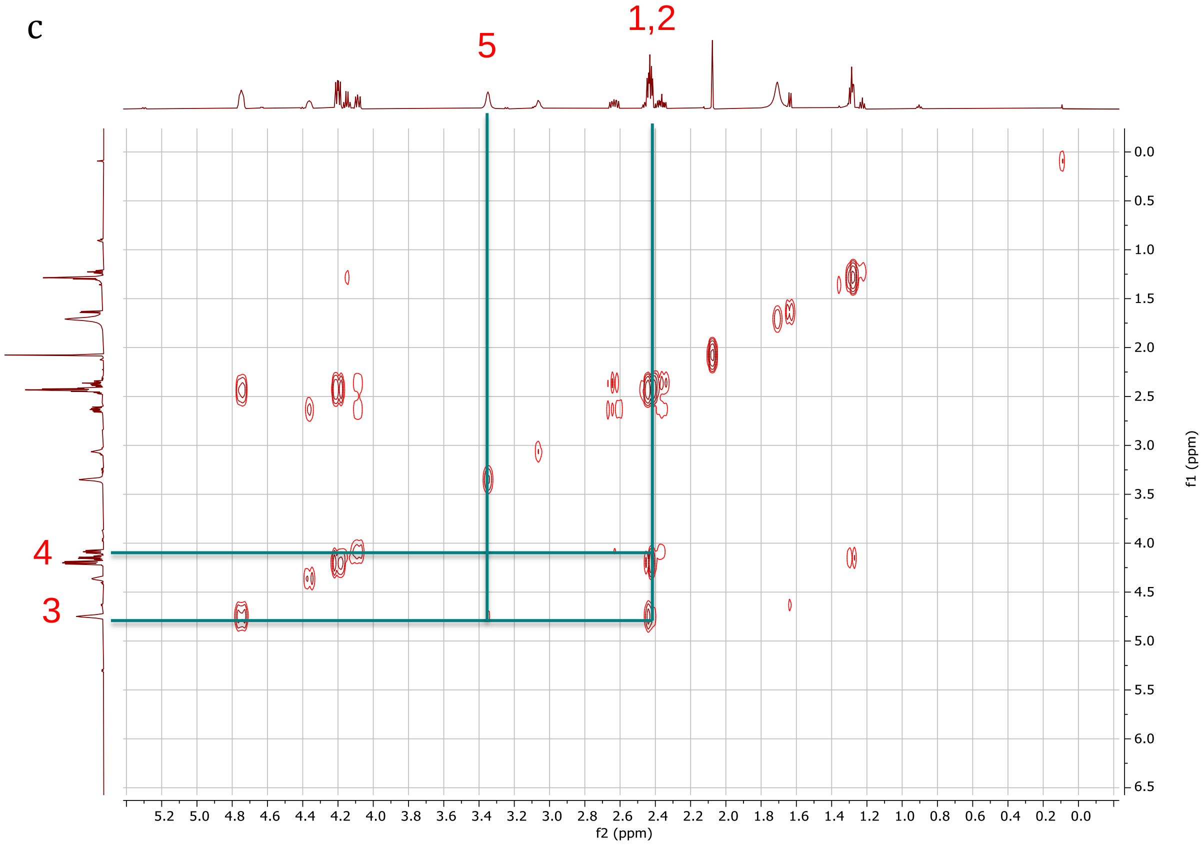
5
1,2
4
3
f2 (ppm)
f1 (ppm)

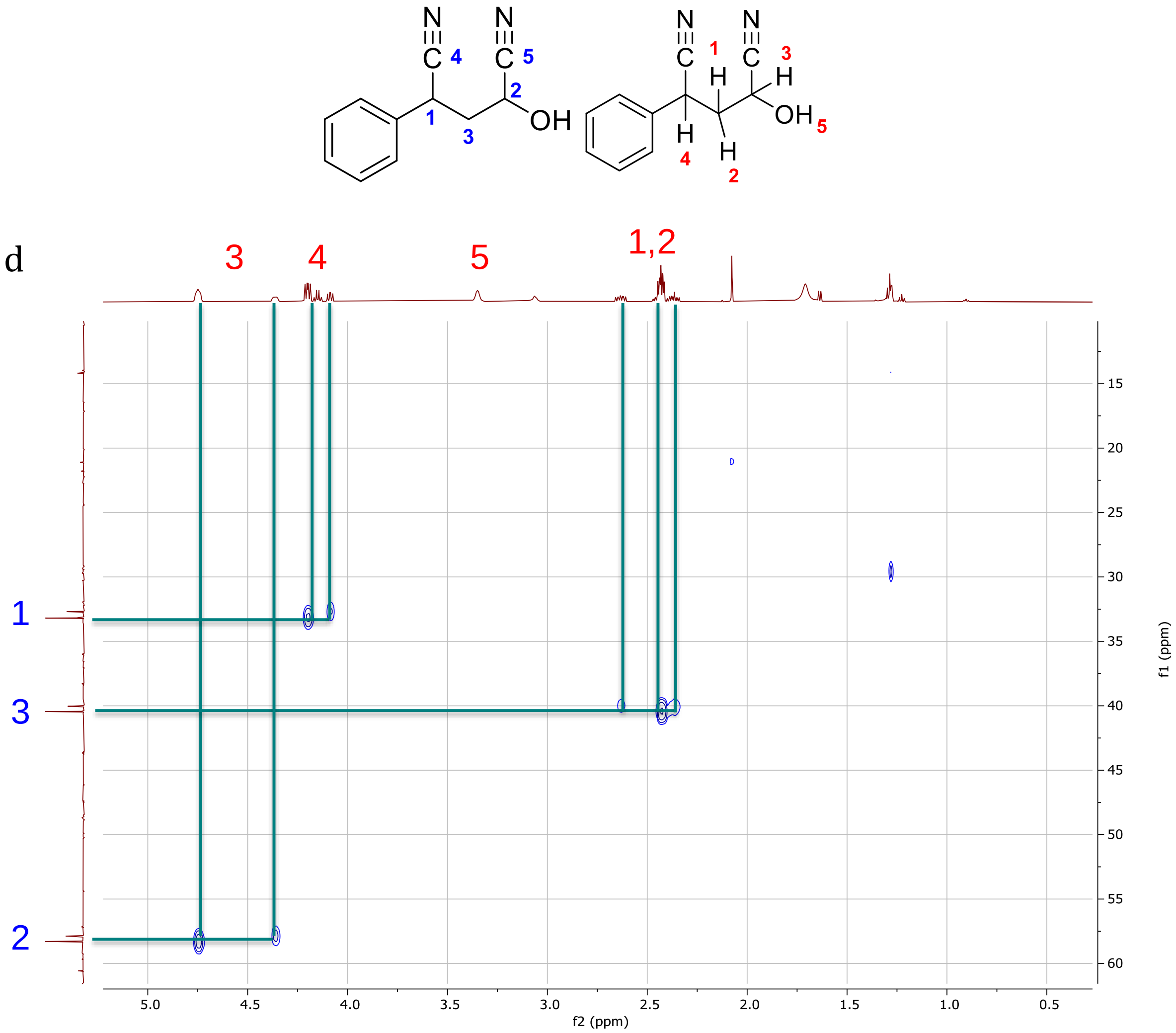

d
3
4
5
1,2
1
3
2
f2 (ppm)
f1 (ppm)

e

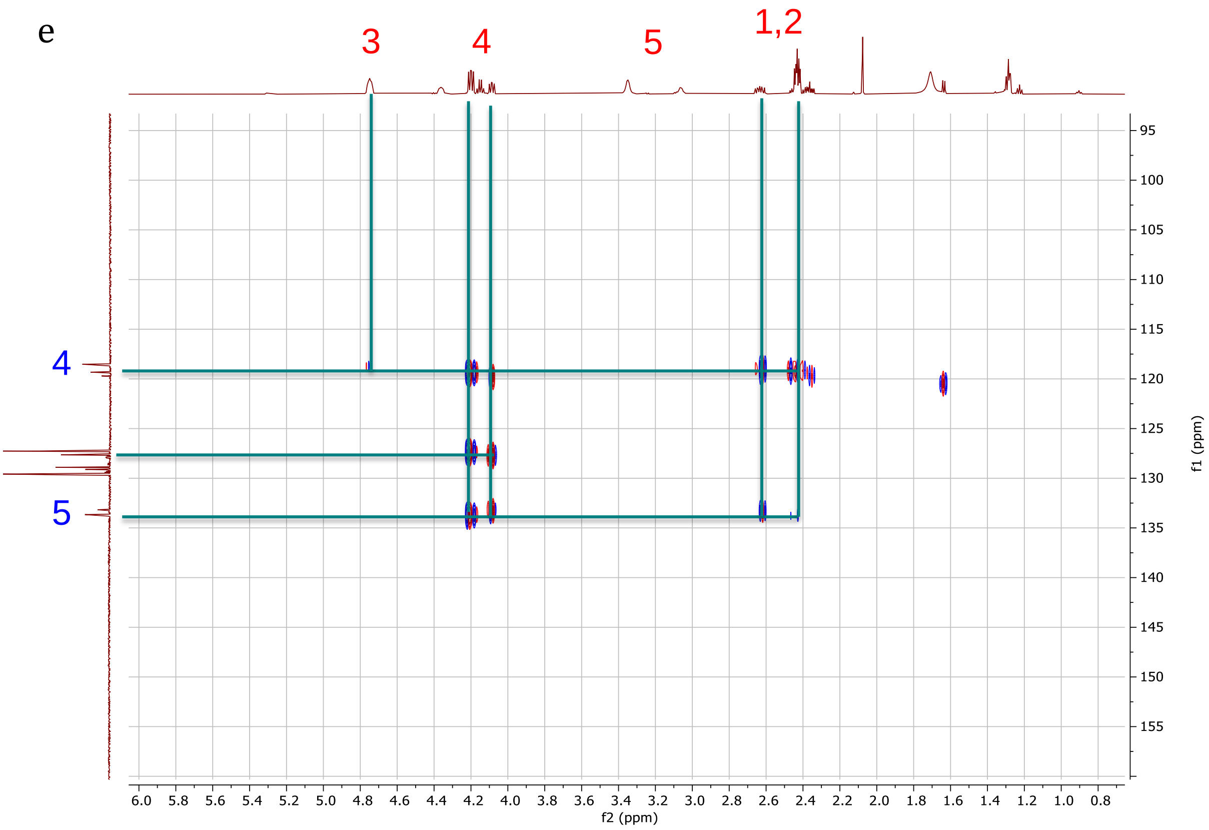

Figure S5. NMR of crude 2-hydroxy-4-phenylpentanedinitrile (**4a**) obtained by upscale synthesis using DERA-EP. a) ^{1}H NMR (600 MHz, $CDCl_{3,}$ mixture of diastereomers, d.r. = 68:32) δ 7.45 – 7.34 (m, 5H), 4.72 (t, *J* = 7.3 Hz, 0.68H), 4.34 (t, *J* = 7.8 Hz, 0.32H)*, 4.18 (dd, *J* = 9.6, 6.3 Hz, 0.74H), 4.06 (dd, *J* = 9.1, 6.6 Hz, 0.34H)*, 3.33 (s, 0.66H), 3.04 (dd, *J* = 6.7, 3.7 Hz, 0.26H)*, 2.61 (ddd, *J* = 13.8, 9.3, 6.7 Hz, 0.35H)*, 2.44 – 2.39 (m, 1.34H), 2.34 (ddd, *J* = 14.0, 9.1, 5.0 Hz, 0.45H)*. * minor diastereomer. b) ^{13}C NMR (151 MHz, $CDCl_3$, mixture of diastereomers) δ 133.79, 133.30, 129.79, 129.70, 129.24, 129.03, 127.78, 127.39, 119.83, 119.45, 58.42, 58.00, 40.58, 40.17, 33.31, 32.83. c) 2D ^{1}H-^{1}H COSY, d) HSQC, e) HMBC.

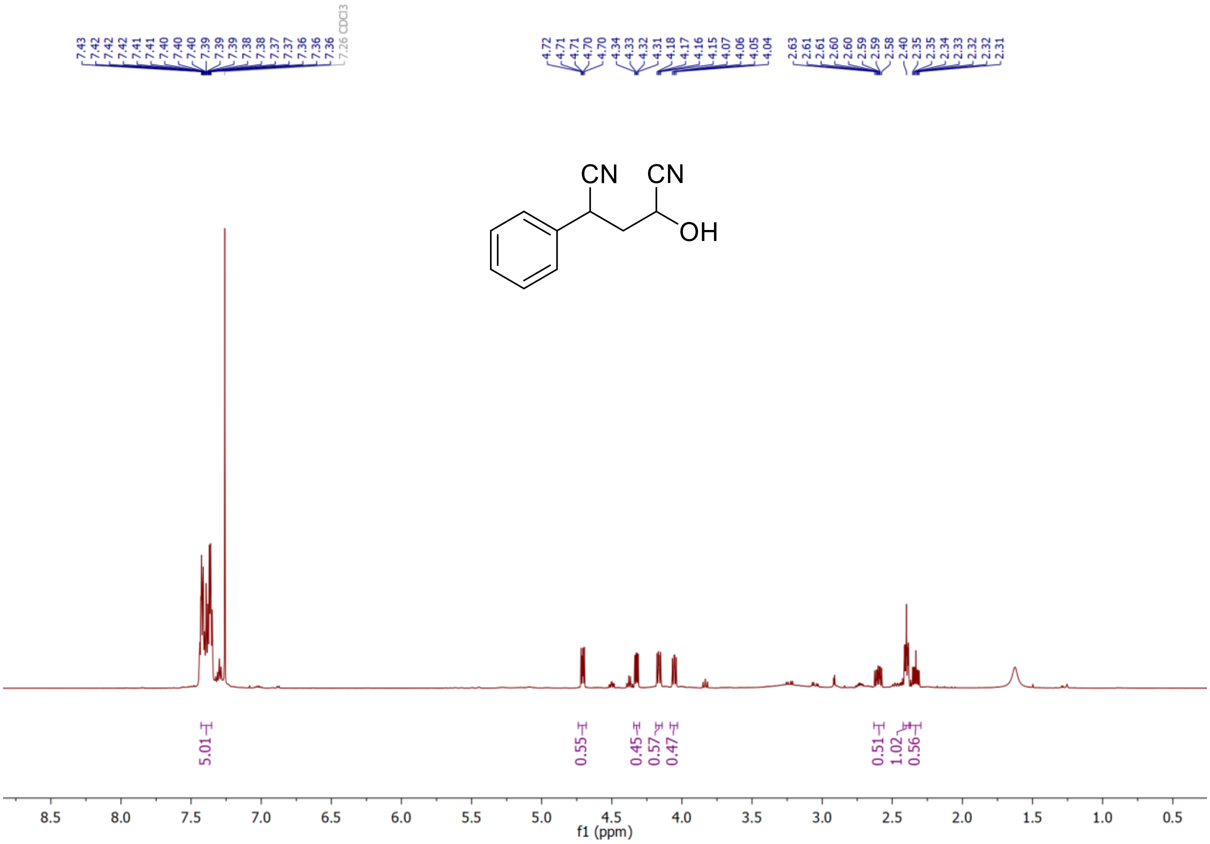

Figure S6. ^{1}H-NMR of *rac*-2-hydroxy-4-phenylpentanedinitrile (**4a**) obtained by chemical synthesis.

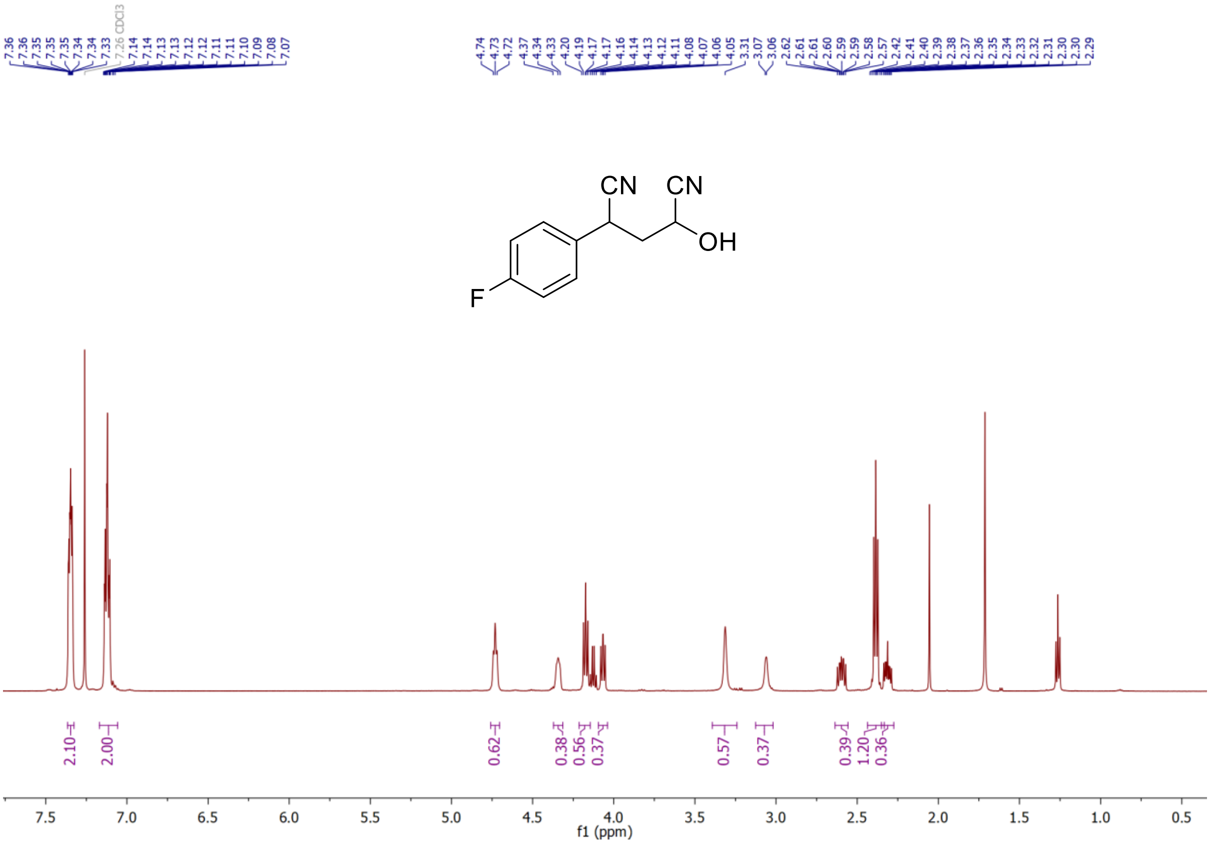

Figure S7. ^{1}H-NMR of *rac*-2-(4-fluorophenyl)-4-hydroxypentanedinitrile (**4b**) obtained by chemical synthesis.

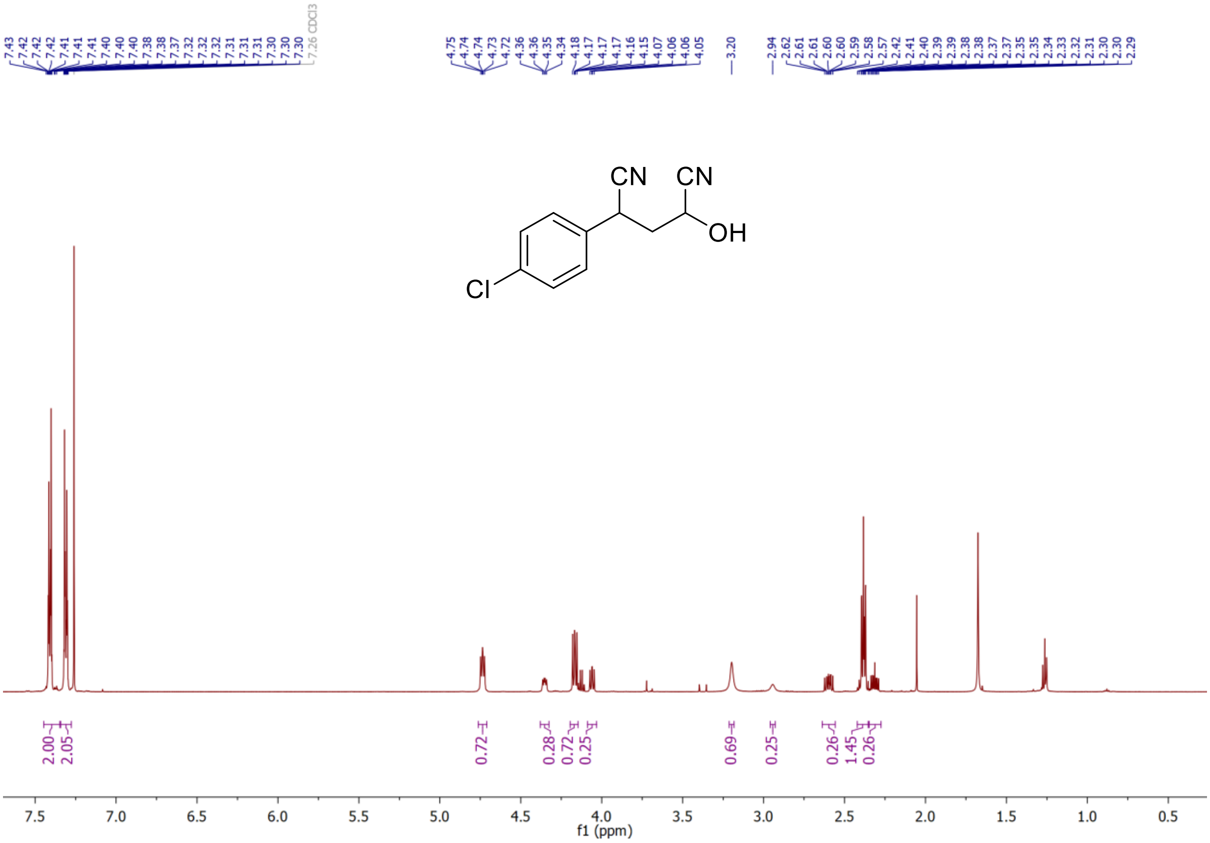

Figure S8. ^{1}H-NMR of *rac*-2-(4-chlorophenyl)-4-hydroxypentanedinitrile (**4c**) obtained by chemical synthesis.

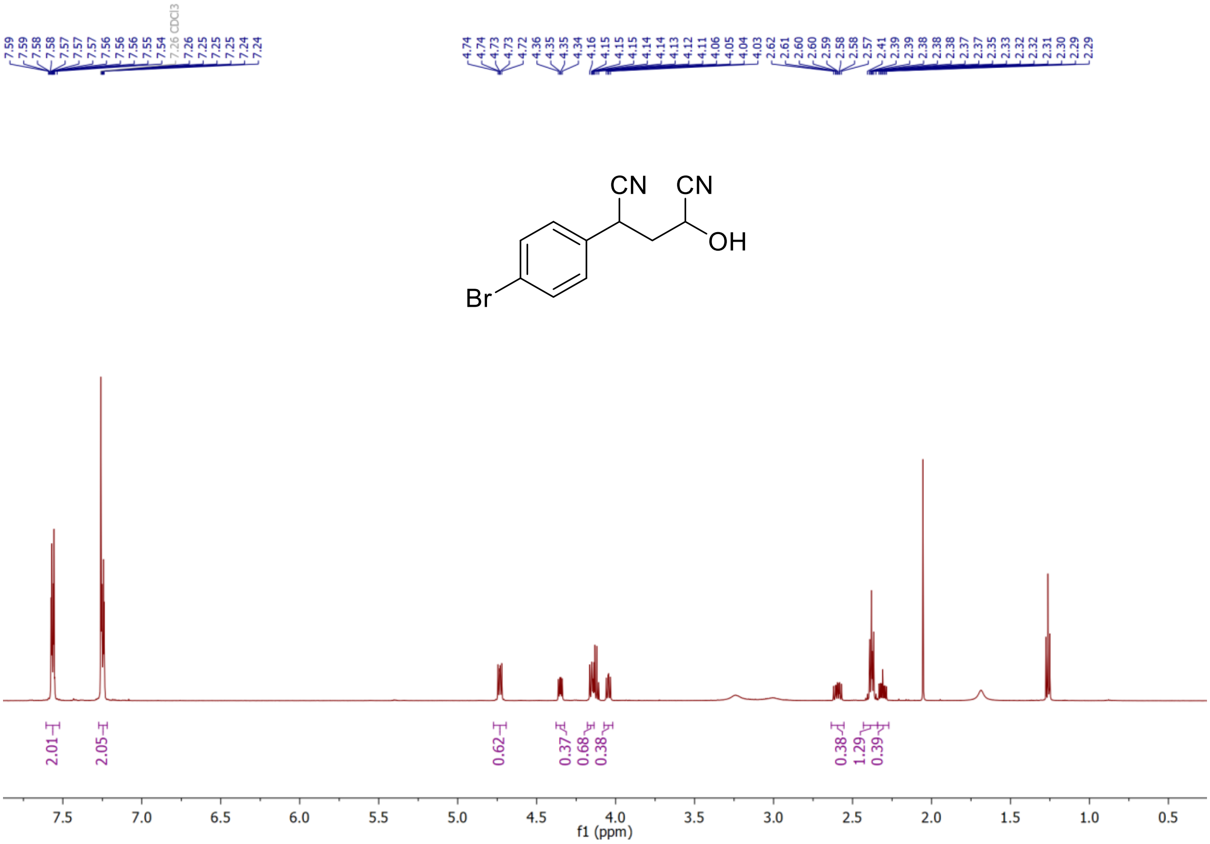

Figure S9. ^{1}H-NMR of *rac*-2-(4-bromophenyl)-4-hydroxypentanedinitrile (**4d**) obtained by chemical synthesis.

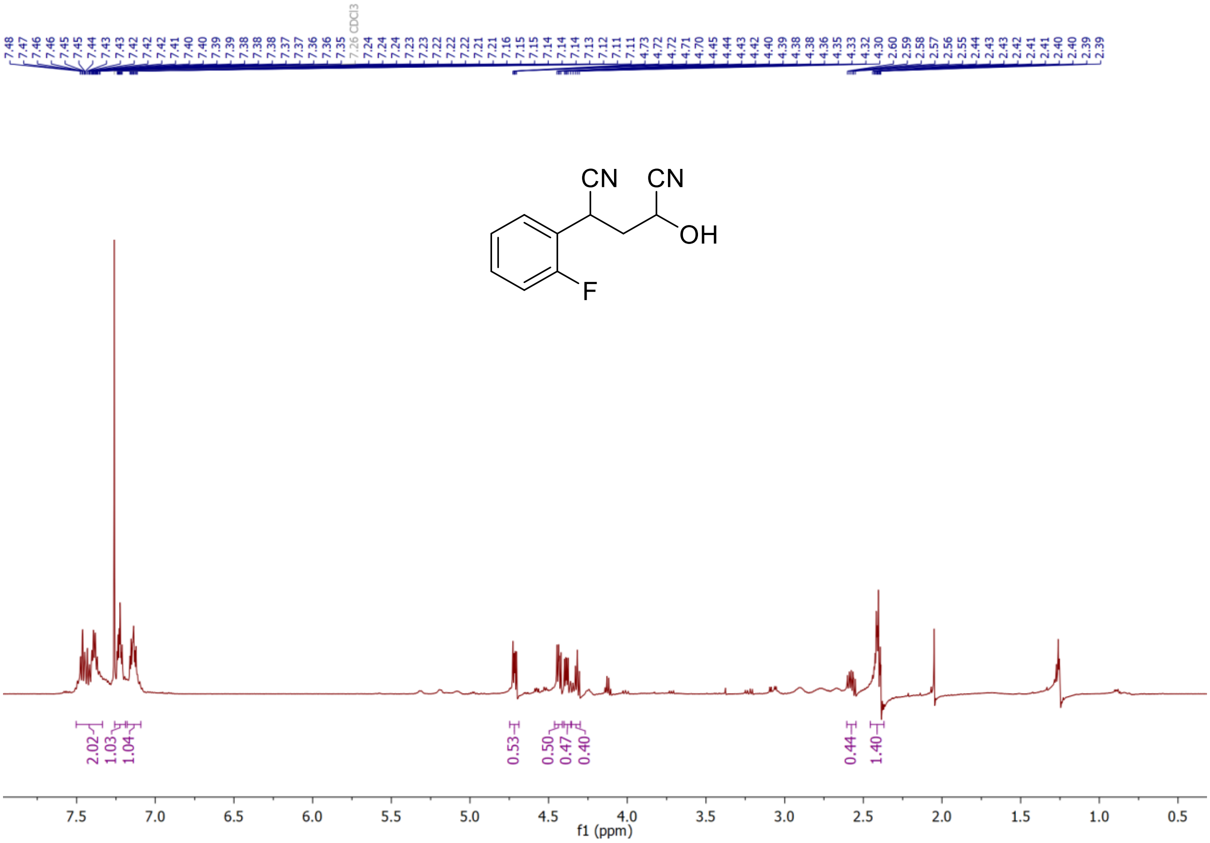

Figure S10. ^{1}H-NMR of *rac*-2-(2-fluorophenyl)-4-hydroxypentanedinitrile (**4e**) obtained by chemical synthesis.

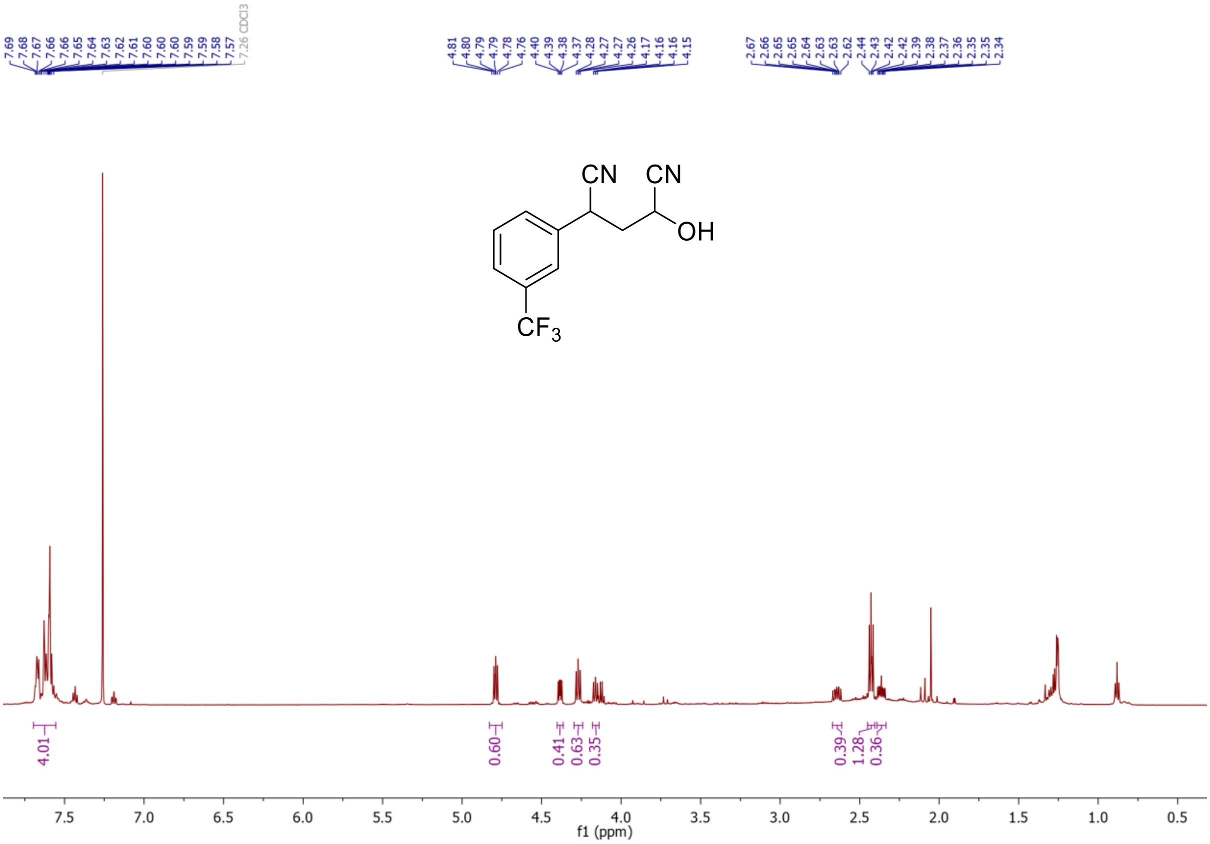

Figure S11. ^{1}H-NMR of *rac*-2-hydroxy-4-(3-(trifluoromethyl)phenyl)pentanedinitrile (**4f**) obtained by chemical synthesis.

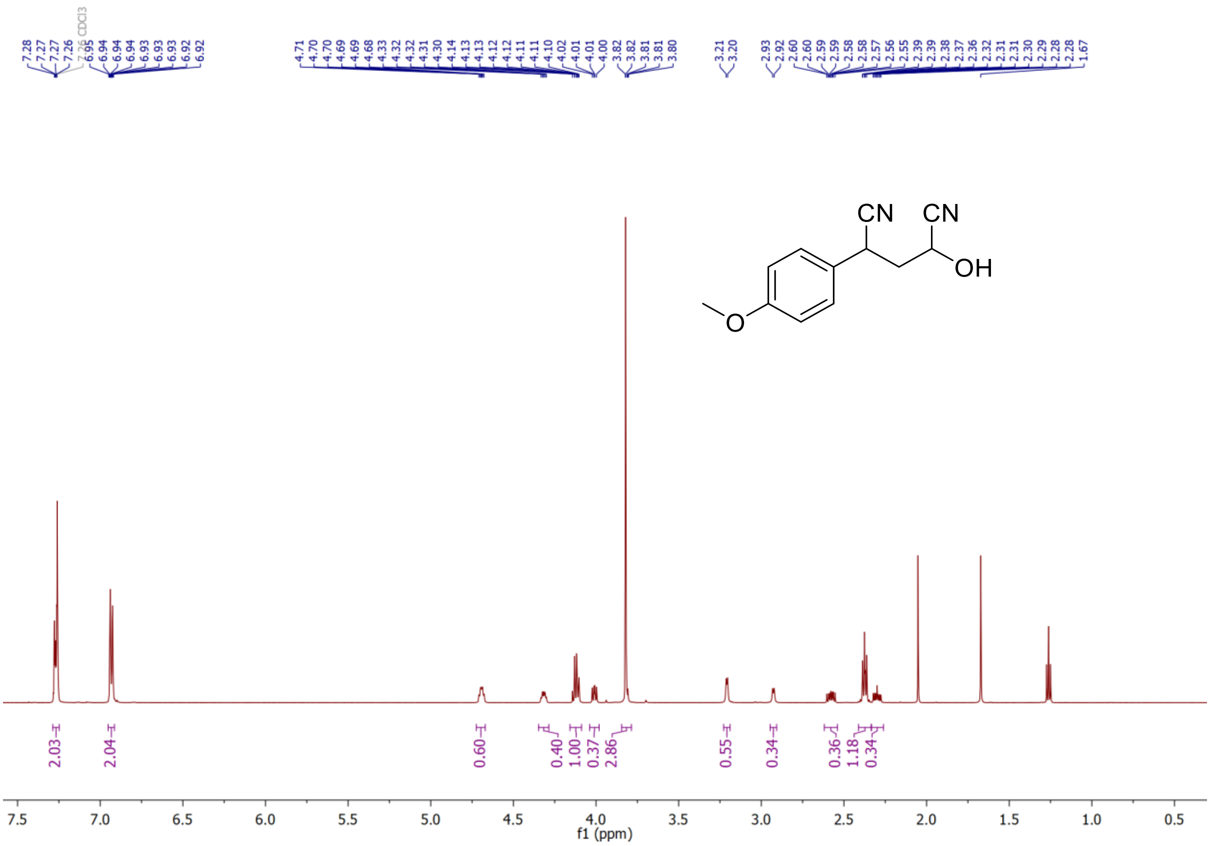

Figure S12. ^{1}H-NMR of *rac*-2-hydroxy-4-(4-methoxyphenyl)pentanedinitrile (**4g**) obtained by chemical synthesis.

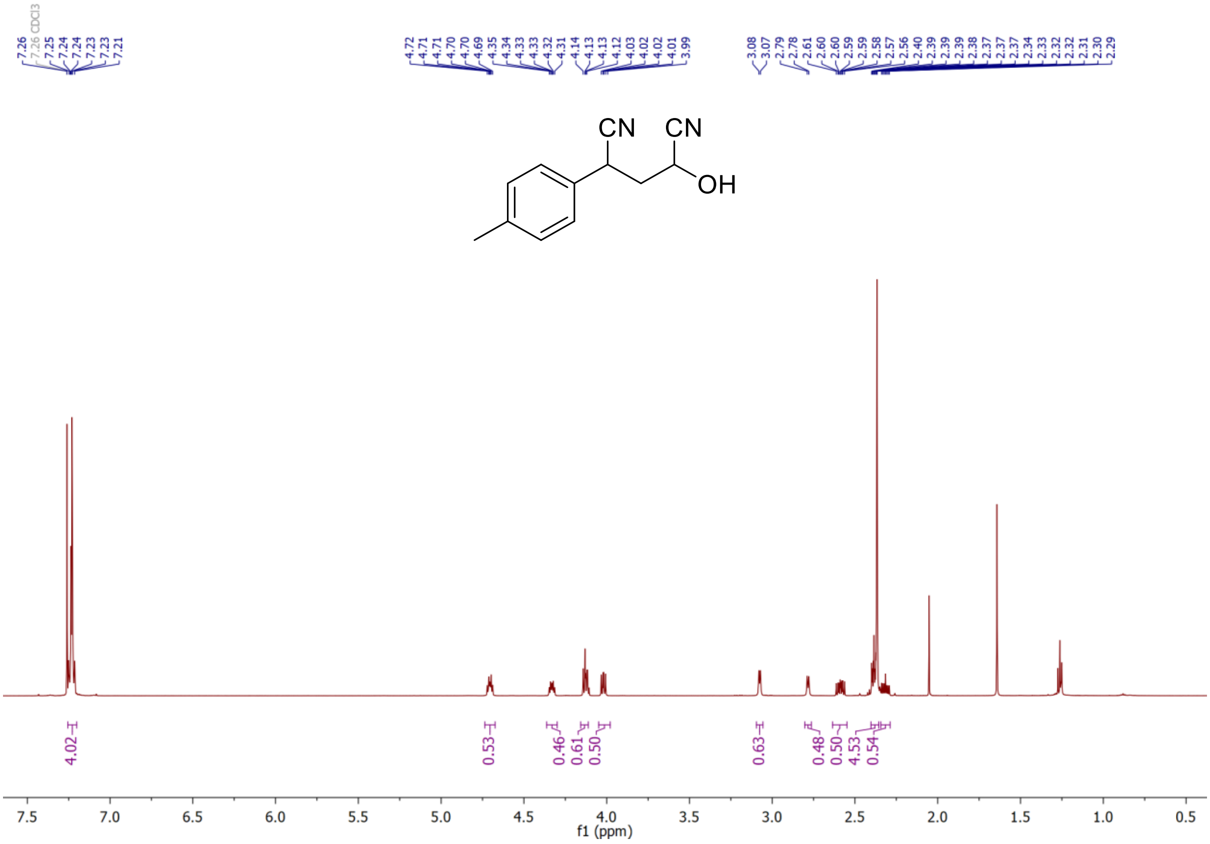

Figure S13. ^{1}H-NMR of *rac*-2-hydroxy-4-(*p*-tolyl)pentanedinitrile (**4h**) obtained by chemical synthesis.

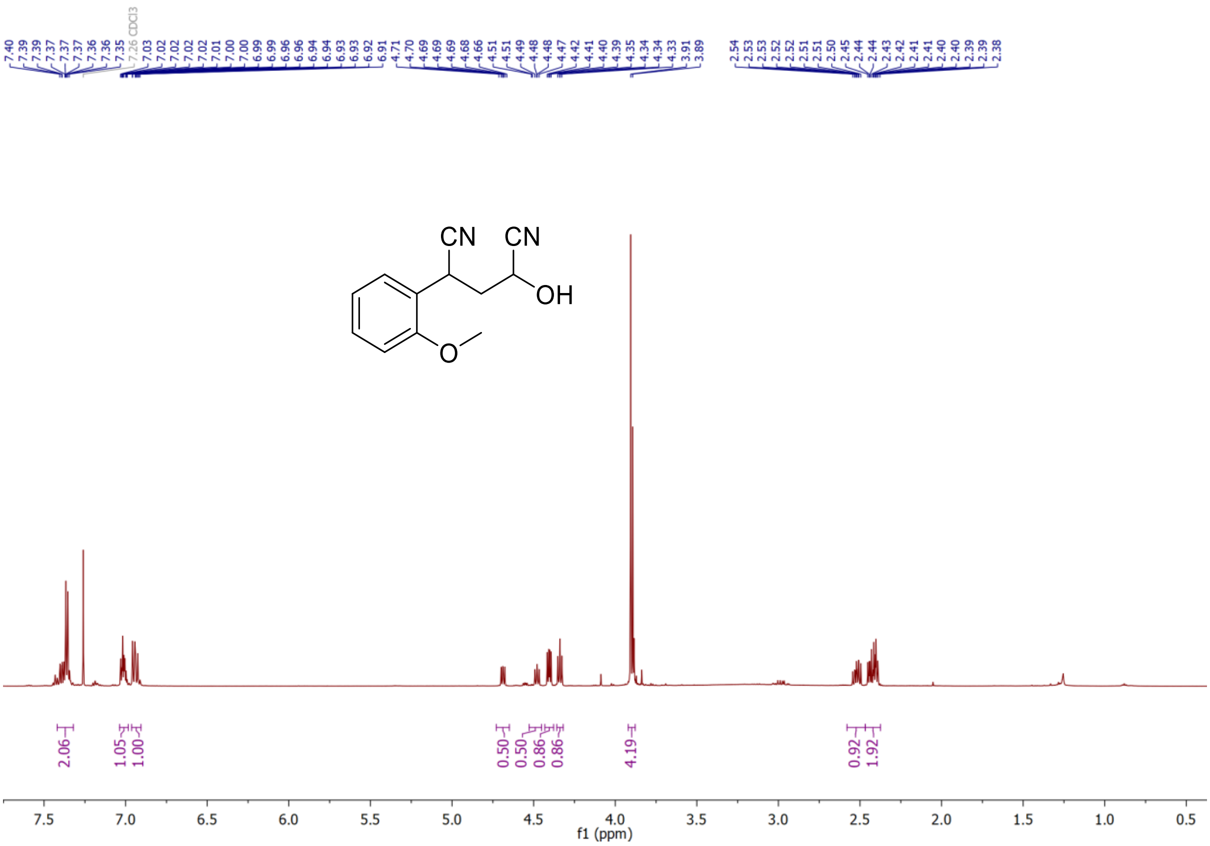

Figure S14. ^{1}H-NMR of *rac*-2-hydroxy-4-(2-methoxyphenyl)pentanedinitrile (**4i**) obtained by chemical synthesis.

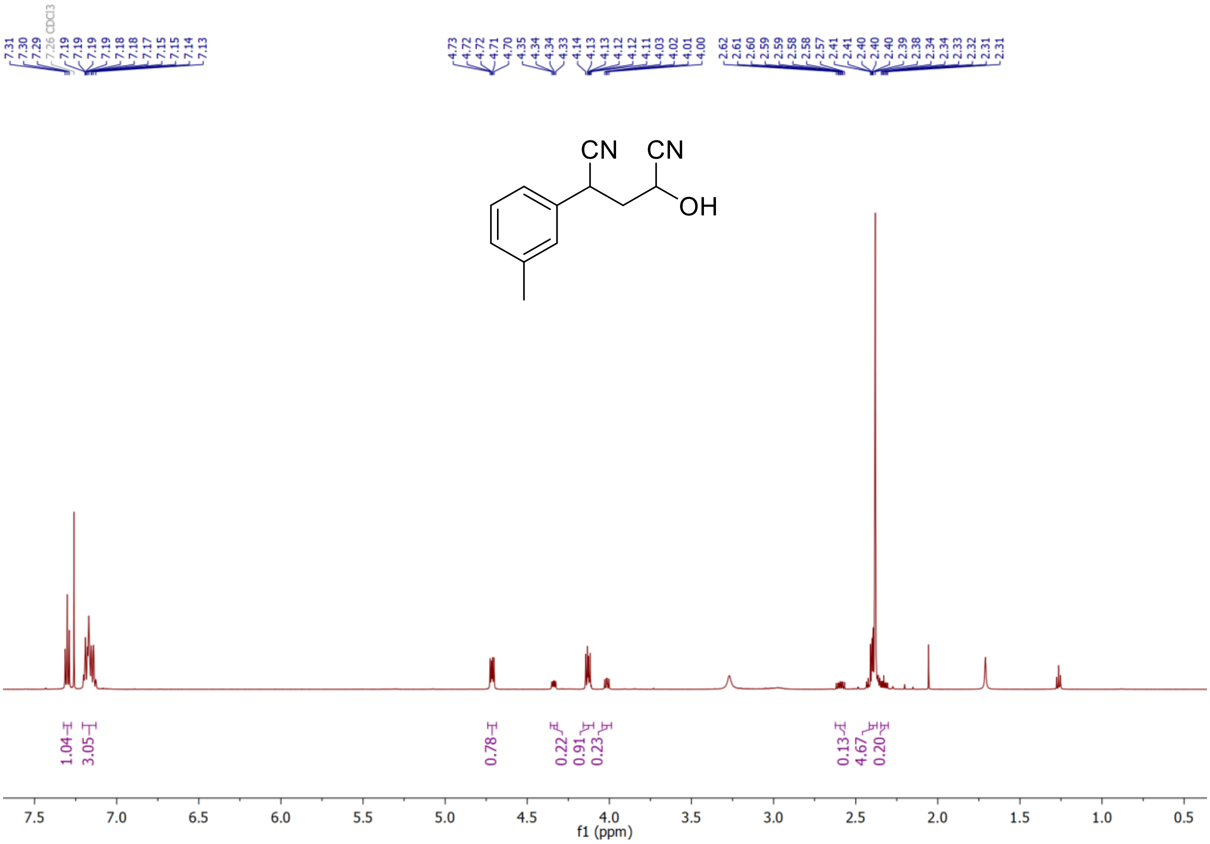

Figure S15. ^{1}H-NMR of *rac*-2-hydroxy-4-(*m*-tolyl)pentanedinitrile (**4j**) obtained by chemical synthesis.

16. GC-FID analysis

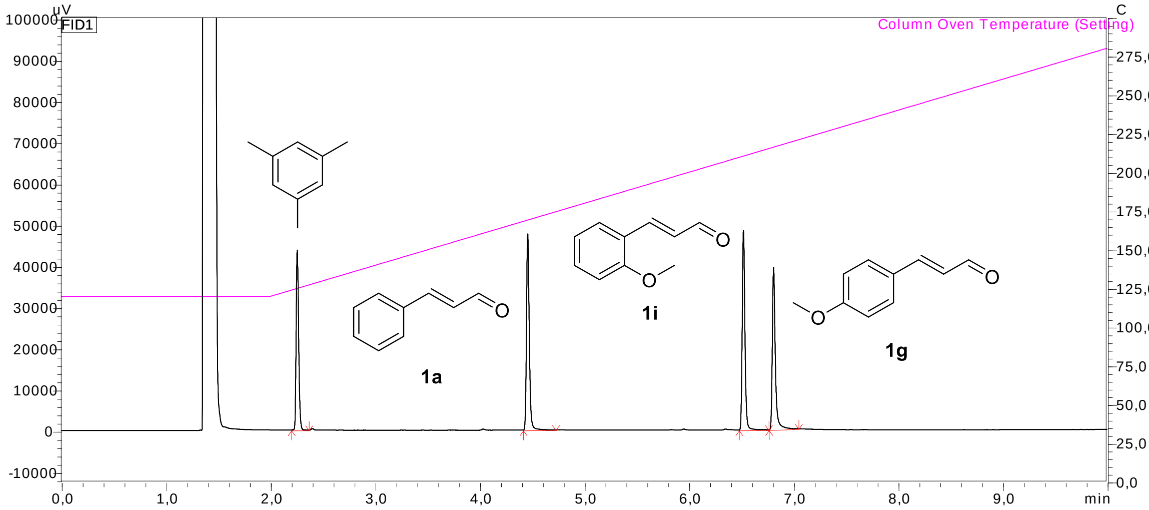

Figure S16. Representative GC-FID chromatogram of **1a**, **1g**, **1i** with internal standard mesitylene.

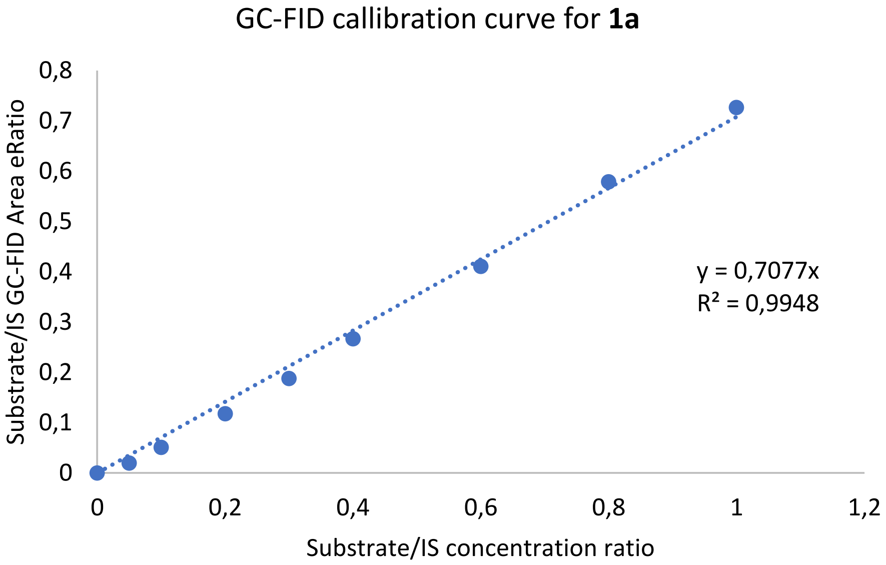

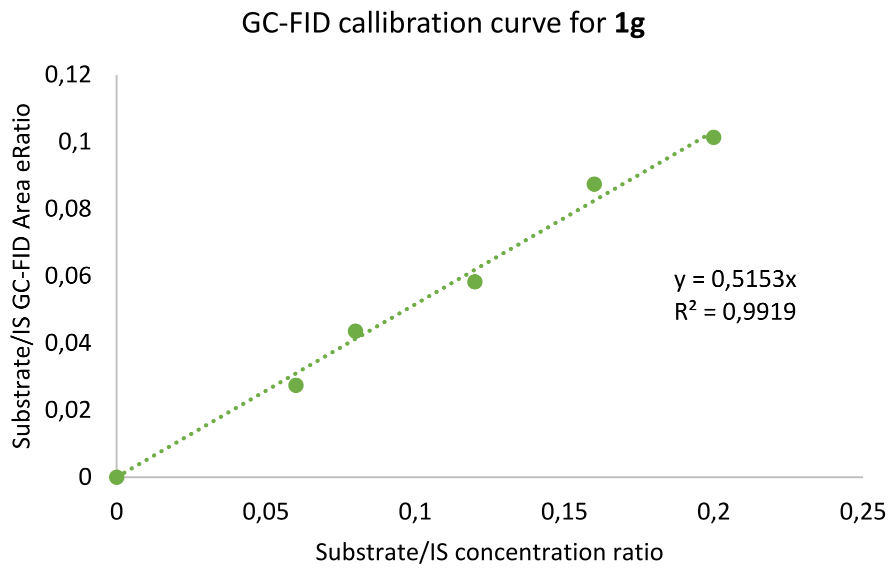

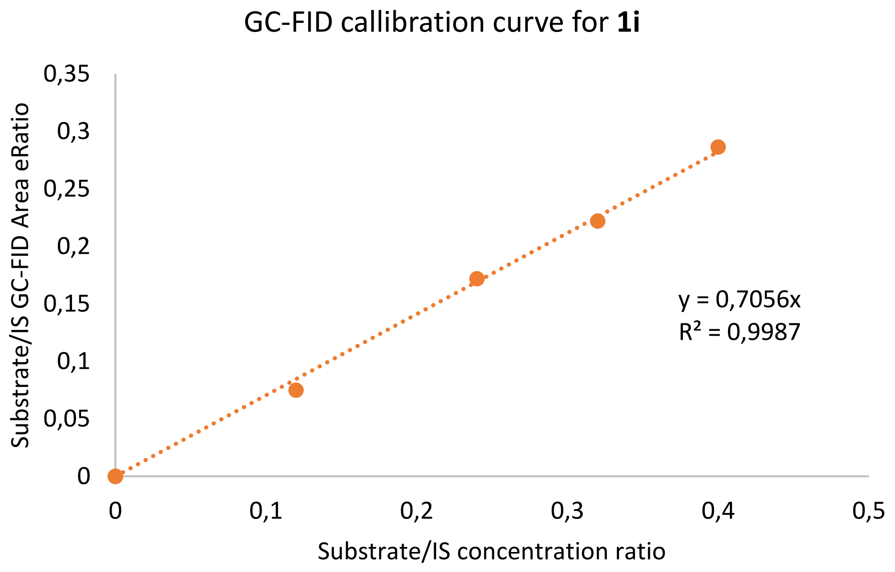

Figure S17. Calibration curves of substrates 1a, 1g and 1i. GC-FID calibration curves were obtained from 1 mM solutions of the internal standard mesitylene in ethyl acetate with different concentrations of the corresponding substrate **1**.

17. Chiral normal-phase HPLC analysis

Table S9. Normal-phase chiral HPLC method and results summary.

Entry	Compound	Column[b]	Mobile phase[c]	Retention time[e] (min)			
				stereoisomer 1	stereoisomer 2	stereoisomer 3	stereoisomer 4
1	**4a**	IC	5%IPA, 95%Hexane[d]				
2	**4b**	ID	10%IPA, 90%Heptane	4.6	5.9	4.9	8.3
3	**4c**	ID	5%IPA, 95%Heptane	8.1	11.4	9.1	19.6
4	**4d**	ID	5%IPA, 95%Heptane	8.8	12.2	9.7	20.5
5	**4e**	IC	10%IPA, 90%Heptane	9.3	23.4	11	20.2
6	**4f**	IC	10%IPA, 90%Heptane	6.4	13.9	7.6	12.1
7	**4g**	ID	10%IPA, 90%Heptane	7.0	8.8	7.8	11.6
8	**4h**	ID	10%IPA, 90%Heptane	4.7	6	5.2	9.2
9	**4i**	OD-H	10%IPA, 90%Heptane	9.8	18.8	11.2	15.6
10	**4j**	IC	10%IPA, 90%Heptane	6.4	13.9	7.6	12.1

[a] UV detection at 210 nm. [b] Column information: Daicel CHIRALPAK® IC (150 × 4.6 mm, 5 µm), CHIRALPAK® ID (150 × 4.6 mm, 5 µm) and CHIRALPAK® OD-H (150 × 4.6 mm, 5 µm); column temperature = 30 °C. [c] Isocratic; flow = 1 mL/min. [d] Isocratic; flow = 1.5 mL/min. [e] Numbers in the tables are from racemic product samples.

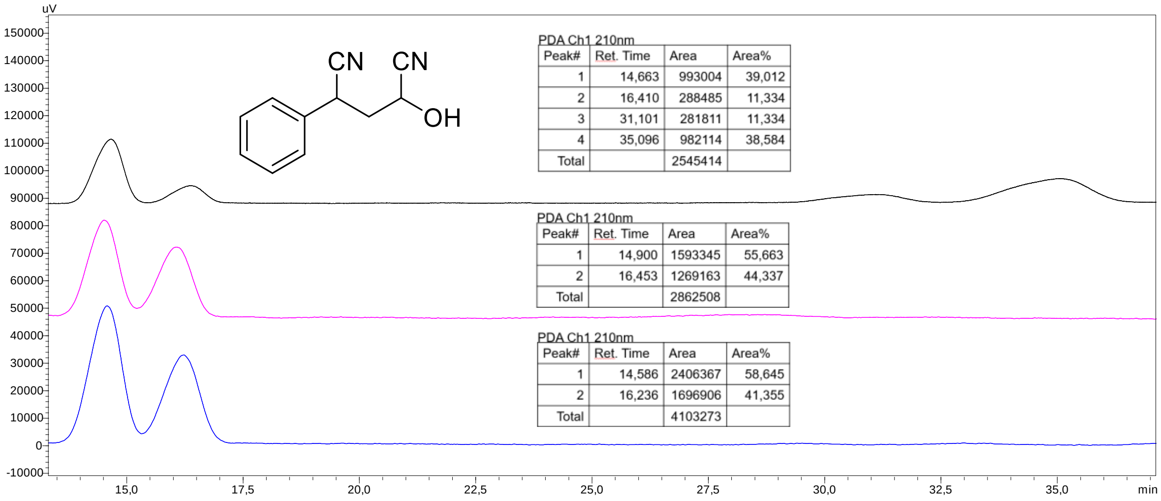

Figure S18. Chiral normal-phase HPLC chromatogram of product 4a. From top to bottom are as follows: chemically synthesized racemic product; enzymatic product generated with DERA-CN in 50 mM citrate (pH 5.5), enzymatic product generated with DERA-CN and *At*HNL in 50 mM citrate (pH 5.5).

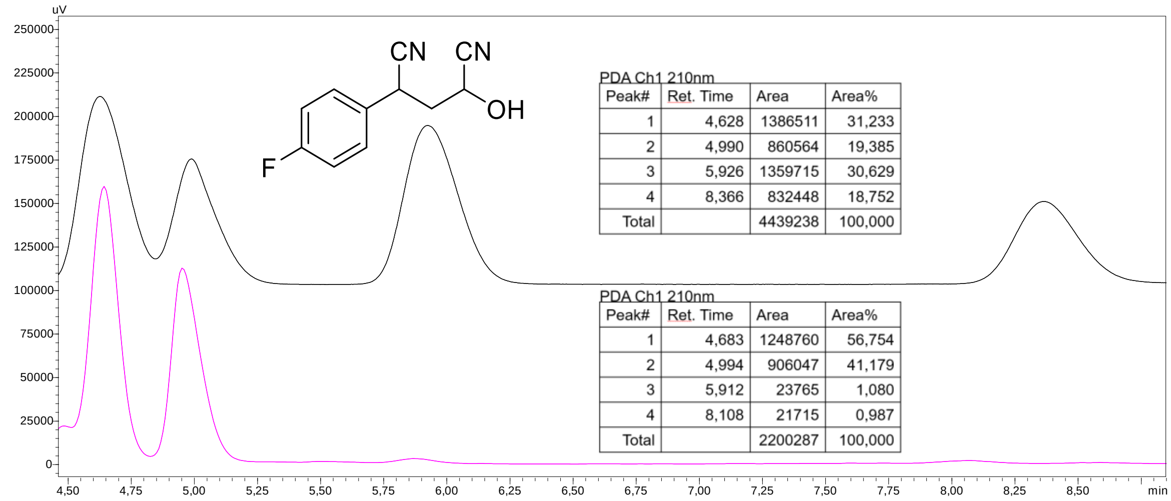

Peak#	Ret. Time	Area	Area%
1	4,628	1386511	31,233
2	4,990	860564	19,385
3	5,926	1359715	30,629
4	8,366	832448	18,752
Total		4439238	100,000

Peak#	Ret. Time	Area	Area%
1	4,683	1248760	56,754
2	4,994	906047	41,179
3	5,912	23765	1,080
4	8,108	21715	0,987
Total		2200287	100,000

Figure S19. Chiral normal-phase HPLC chromatogram of product 4b. From top to bottom are as follows: chemically synthesized racemic product and enzymatic product generated with DERA-CN in 50 mM citrate (pH 5.5).

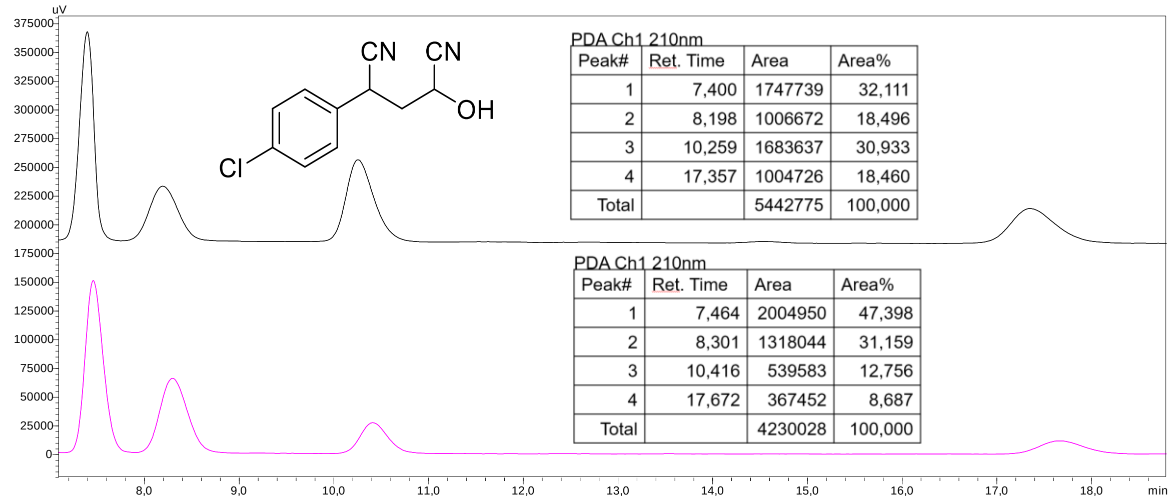

Peak#	Ret. Time	Area	Area%
1	7,400	1747739	32,111
2	8,198	1006672	18,496
3	10,259	1683637	30,933
4	17,357	1004726	18,460
Total		5442775	100,000

Peak#	Ret. Time	Area	Area%
1	7,464	2004950	47,398
2	8,301	1318044	31,159
3	10,416	539583	12,756
4	17,672	367452	8,687
Total		4230028	100,000

Figure S20. Chiral normal-phase HPLC chromatogram of product 4c. From top to bottom are as follows: chemically synthesized racemic product and enzymatic product generated with DERA-CN in 50 mM citrate (pH 5.5).

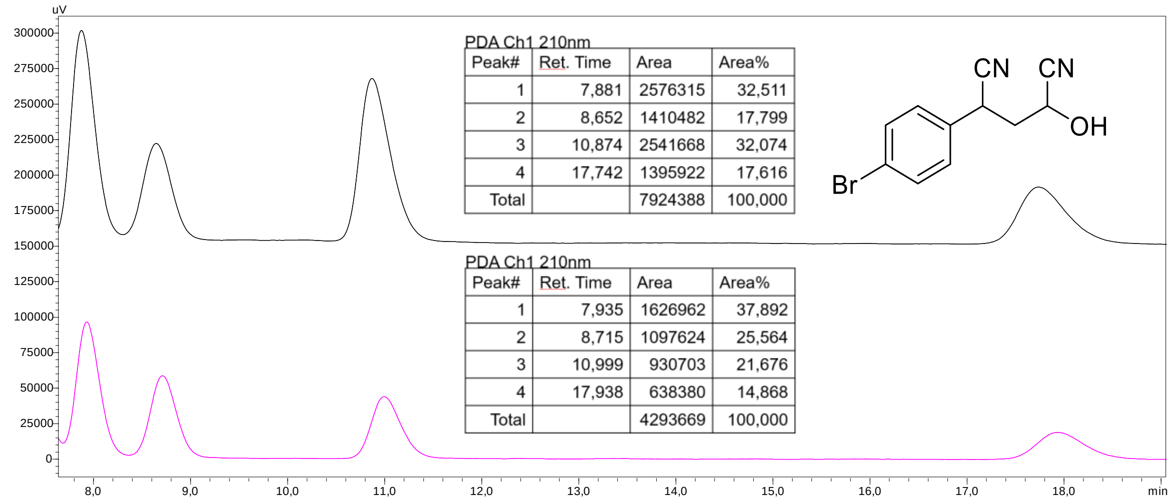

PDA Ch1 210nm

Peak#	Ret. Time	Area	Area%
1	7,881	2576315	32,511
2	8,652	1410482	17,799
3	10,874	2541668	32,074
4	17,742	1395922	17,616
Total		7924388	100,000

PDA Ch1 210nm

Peak#	Ret. Time	Area	Area%
1	7,935	1626962	37,892
2	8,715	1097624	25,564
3	10,999	930703	21,676
4	17,938	638380	14,868
Total		4293669	100,000

Figure S21. Chiral normal-phase HPLC chromatogram of product 4d. From top to bottom are as follows: chemically synthesized racemic product and enzymatic product generated with DERA-CN in 50 mM citrate (pH 5.5).

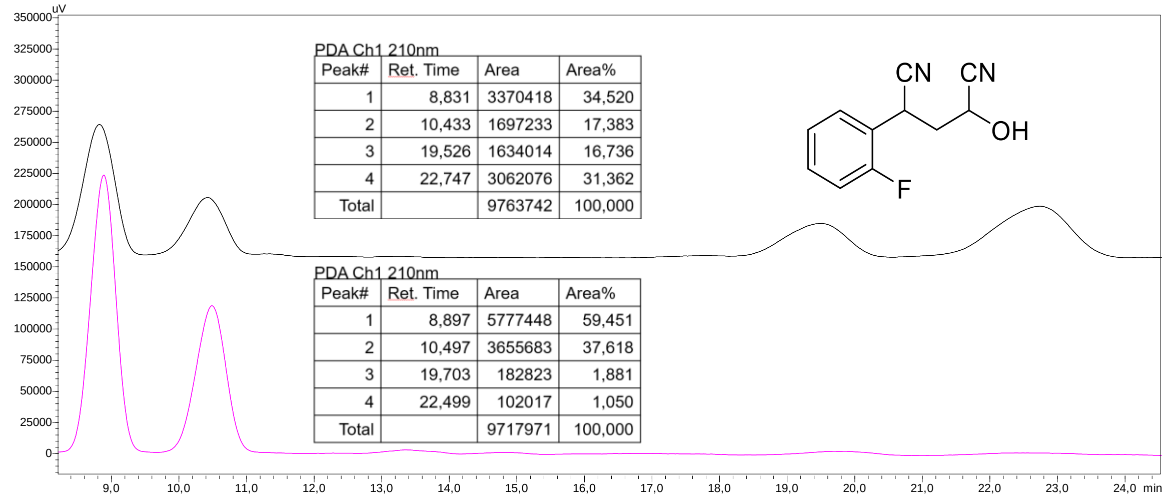

PDA Ch1 210nm

Peak#	Ret. Time	Area	Area%
1	8,831	3370418	34,520
2	10,433	1697233	17,383
3	19,526	1634014	16,736
4	22,747	3062076	31,362
Total		9763742	100,000

PDA Ch1 210nm

Peak#	Ret. Time	Area	Area%
1	8,897	5777448	59,451
2	10,497	3655683	37,618
3	19,703	182823	1,881
4	22,499	102017	1,050
Total		9717971	100,000

Figure S22. Chiral normal-phase HPLC chromatogram of product 4e. From top to bottom are as follows: chemically synthesized racemic product and enzymatic product generated with DERA-CN in 50 mM citrate (pH 5.5).

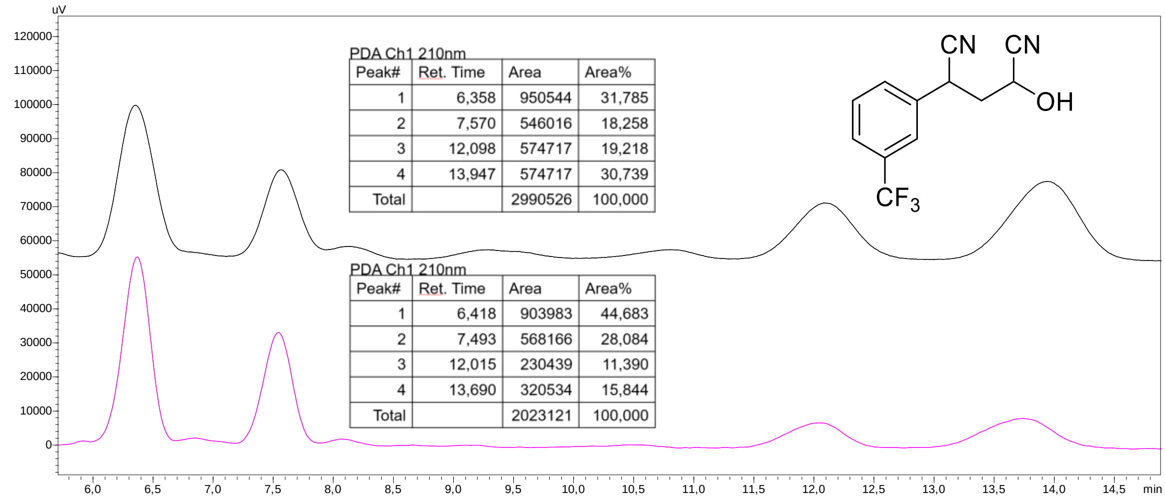

Figure S23. Chiral normal-phase HPLC chromatogram of product 4f. From top to bottom are as follows: chemically synthesized racemic product and enzymatic product generated with DERA-CN in 50 mM citrate (pH 5.5).

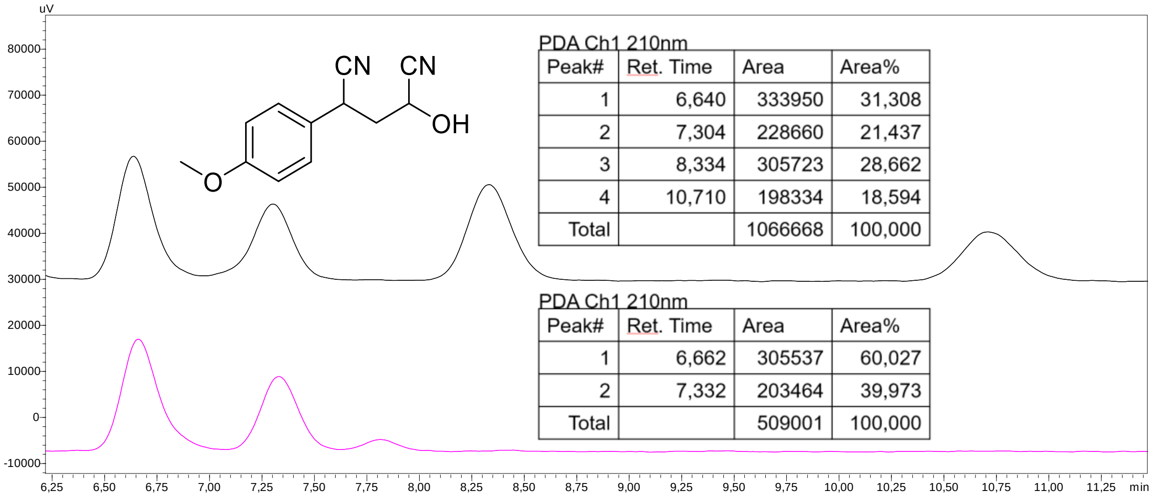

Figure S24. Chiral normal-phase HPLC chromatogram of product 4g. From top to bottom are as follows: chemically synthesized racemic product and enzymatic product generated with DERA-CN in 50 mM citrate (pH 5.5).

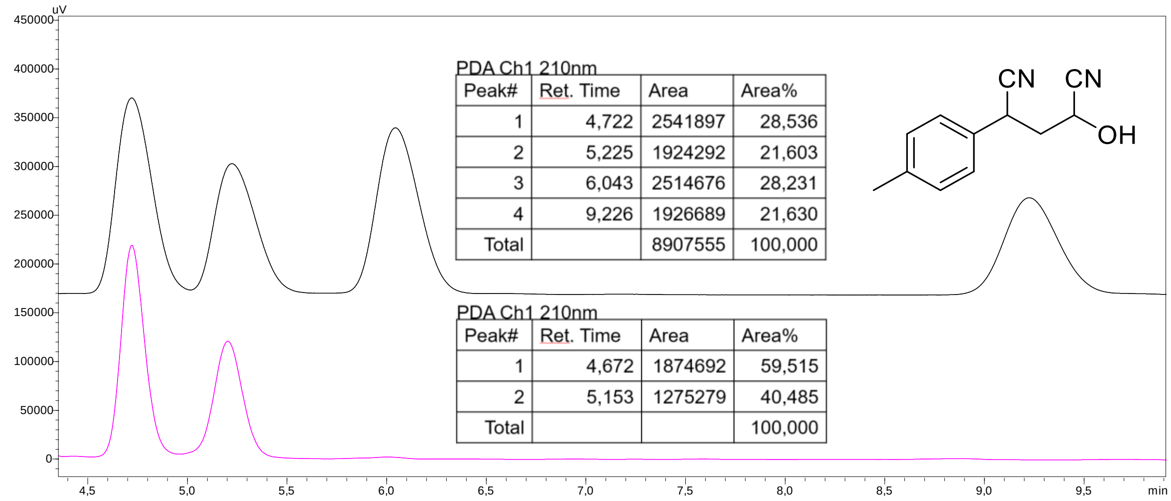

PDA Ch1 210nm

Peak#	Ret. Time	Area	Area%
1	4,722	2541897	28,536
2	5,225	1924292	21,603
3	6,043	2514676	28,231
4	9,226	1926689	21,630
Total		8907555	100,000

PDA Ch1 210nm

Peak#	Ret. Time	Area	Area%
1	4,672	1874692	59,515
2	5,153	1275279	40,485
Total			100,000

Figure S25. Chiral normal-phase HPLC chromatogram of product 4h. From top to bottom are as follows: chemically synthesized racemic product and enzymatic product generated with DERA-CN in 50 mM citrate (pH 5.5).

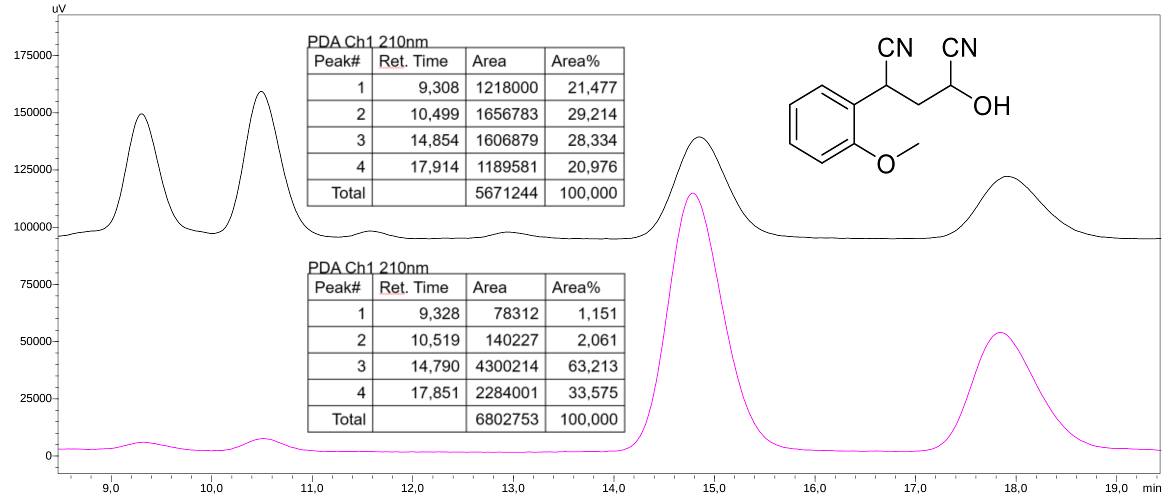

PDA Ch1 210nm

Peak#	Ret. Time	Area	Area%
1	9,308	1218000	21,477
2	10,499	1656783	29,214
3	14,854	1606879	28,334
4	17,914	1189581	20,976
Total		5671244	100,000

PDA Ch1 210nm

Peak#	Ret. Time	Area	Area%
1	9,328	78312	1,151
2	10,519	140227	2,061
3	14,790	4300214	63,213
4	17,851	2284001	33,575
Total		6802753	100,000

Figure S26. Chiral normal-phase HPLC chromatogram of product 4i. From top to bottom are as follows: chemically synthesized racemic product and enzymatic product generated with DERA-CN in 50 mM citrate (pH 5.5).

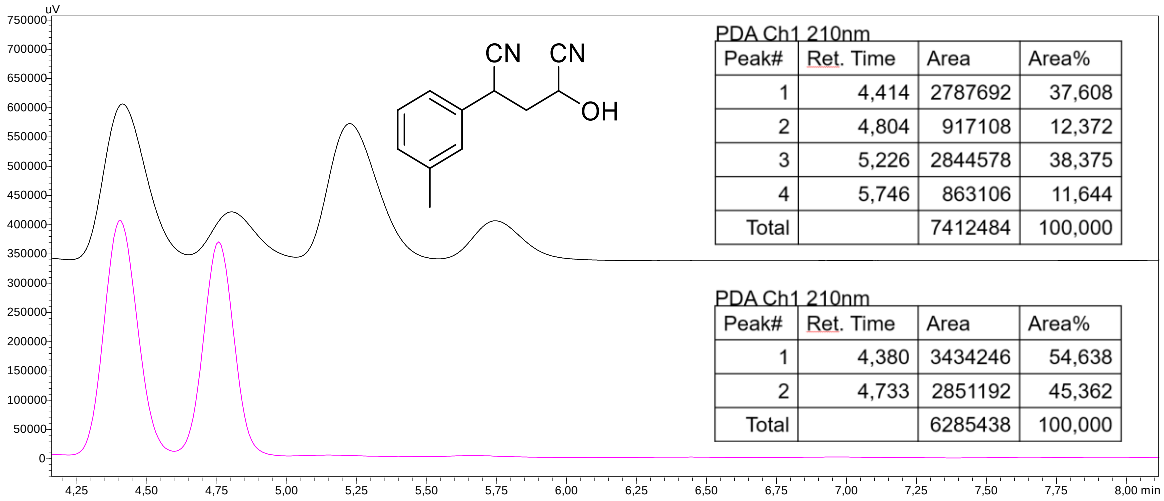

Figure S27. Chiral normal-phase HPLC chromatogram of product 4j. From top to bottom are as follows: chemically synthesized racemic product and enzymatic product generated with DERA-CN in 50 mM citrate (pH 5.5).

18. Supplementary references

[1] A. Kunzendorf, G. Xu, J. J. H. van der Velde, H. J. Rozeboom, A. W. H. Thunnissen, G. J. Poelarends, *ACS Catal.* **2021**, *11*, 13236–13243.
[2] H. Zhou, A. Kunzendorf, G. Xu, H. O. T. Frietema, A. W. H. Thunnissen, G. J. Poelarends, *Angew. Chem. Int. Ed.* **2025**, e202503054.
[3] T. A. Kunkel, *Proc. Natl. Acad. Sci.* **1985**, *82*, 488–492.
[4] D. S. Wilson, A. D. Keefe, *Curr. Protoc. Mol. Biol.* **2000**, *51*, 1–9.
[5] P. C. Cirino, K. M. Mayer, D. Umeno, *Dir. Evol. Libr. Creat.* **2003**, *231*, 3–10.
[6] L. Biewenga, M. Crotti, M. Saifuddin, G. J. Poelarends, *ACS Omega.* **2020**, *5*, 2397–2405.
[7] K. Schultz, M. Hesse, *Tetrahedron* **1996**, *52*, 14189–14198.
[8] J. Fairbanks, *J. Org. Chem.* **1983**, *48*, 4155–4156.
[9] D. B. Dess, J. C. Martin, *J. Am. Chem. Soc.* **1991**, *113*, 7277–7287.
[10] S. Kobayashi, Y.Tsuchiya, T. Mukaiyama *Chem. Lett.* **1991**, 537–540.
